# A systematic evaluation of normalization methods and probe replicability using infinium EPIC methylation data

**DOI:** 10.1186/s13148-023-01459-z

**Published:** 2023-03-11

**Authors:** H. Welsh, C. M. P. F. Batalha, W. Li, K. L. Mpye, N. C. Souza-Pinto, M. S. Naslavsky, E. J. Parra

**Affiliations:** 1 Department of Anthropology, University of Toronto at Mississauga, Mississauga, Canada; 2 Department of Biochemistry, University of São Paulo, São Paulo, Brazil; 3 The Centre for Applied Genomics, Hospital for Sick Children, Toronto, Canada; 4 Department of Genetics and Evolutionary Biology, University of São Paulo, São Paulo, Brazil

**Keywords:** DNA methylation, Illumina EPIC array, Normalization, Reproducibility, ICC

## Abstract

**Background:**

The Infinium EPIC array measures the methylation status of > 850,000 CpG sites. The EPIC BeadChip uses a two-array design: Infinium Type I and Type II probes. These probe types exhibit different technical characteristics which may confound analyses. Numerous normalization and pre-processing methods have been developed to reduce probe type bias as well as other issues such as background and dye bias.

**Methods:**

This study evaluates the performance of various normalization methods using 16 replicated samples and three metrics: absolute beta-value difference, overlap of non-replicated CpGs between replicate pairs, and effect on beta-value distributions. Additionally, we carried out Pearson’s correlation and intraclass correlation coefficient (ICC) analyses using both raw and SeSAMe 2 normalized data.

**Results:**

The method we define as SeSAMe 2, which consists of the application of the regular SeSAMe pipeline with an additional round of QC, pOOBAH masking, was found to be the best performing normalization method, while quantile-based methods were found to be the worst performing methods. Whole-array Pearson’s correlations were found to be high. However, in agreement with previous studies, a substantial proportion of the probes on the EPIC array showed poor reproducibility (ICC < 0.50). The majority of poor performing probes have beta values close to either 0 or 1, and relatively low standard deviations. These results suggest that probe reliability is largely the result of limited biological variation rather than technical measurement variation. Importantly, normalizing the data with SeSAMe 2 dramatically improved ICC estimates, with the proportion of probes with ICC values > 0.50 increasing from 45.18% (raw data) to 61.35% (SeSAMe 2).

**Supplementary Information:**

The online version contains supplementary material available at 10.1186/s13148-023-01459-z.

## Introduction

Epigenetic mechanisms, such as DNA methylation, are known to regulate gene function and phenotypic expression [1–3]. Variation in DNA methylation is associated with premature aging and a variety of age-related illnesses including cancer, diabetes, cardiovascular disease, metabolic disease, and neurological diseases [4, 5]. Therefore, analysing DNA methylation can provide important information regarding disease risk and health outcomes.

Whole-genome bisulphite sequencing (WGBS) is the *gold standard* for sequencing and mapping CpG methylation [6]. However, due to lower costs, low input DNA requirements and high-throughput capabilities, array-based methods for assessing DNA methylation have been widely used. The Illumina DNA methylation BeadChips (HumanMethylation27, HumanMethylation450, HumanMethylationEPIC) have been the most popular high-density microarrays for methylation studies [7, 8]. The Infinium HumanMethylationEPIC (EPIC) array, released in 2015, is the most recent and robust Illumina microarray [9]. Illumina’s EPIC array is designed to assess methylation levels at 863,904 CpG sites [10].

The Illumina microarrays are built on the same technology used for genotyping single-nucleotide polymorphisms (SNPs) [10]. The Illumina HumanMethylation450k (450k) and EPIC BeadChips use a two-array design: Infinium Type I probes and Infinium Type II probes. Type I probes have two beads per CpG site, the first to measure the methylated intensity and the second to measure the unmethylated intensity [9, 11]. Type II probes use a single bead to interrogate each CpG site. This bead only measures methylated intensity [11]. The intensities are used to determine the proportion of methylation at each CpG site, which are reported as either beta (*β*) values or logit transformed *M*-values [10, 11].

The accuracy of certain probes and methylation output can be influenced by confounding factors such as the presence of SNPs, probe cross-reactivity, cell heterogeneity, between-array biases (batch effects), and within-array biases [9, 10]. Within-array biases can include background, dye bias, and probe-type bias [9]. Correcting probe-type bias is especially critical as it is the main source of decreasing data quality [9, 12] and occurs because Infinium Type I and Type II probes differ in their design and produce different beta-value distributions [7, 13]. However, various pre-processing and normalization tools are available to increase output reliability [14].

Normalization procedures currently available for correcting EPIC array methylation output include Quantile normalization (QN) [15], beta-mixture quantile normalization (BMIQ) [16], subset-quantiles within microarray normalization (SWAN) [17], peak-based correction (PBC) [18], functional normalization (Funnorm) [19], normal-exponential convolution using out-of-band probes (Noob) [20], single-sample noob (SSnoob) [21], and sensible step-wise analysis of DNA methylation BeadChips (SeSAMe) [22]. While there are a variety of normalization options for methylation data, BMIQ is the most widely used method for correcting probe distributions [7]. Some studies comparing different normalization methods using the 450k array did not find significant differences in the overall results [13, 23]. However, when comparing normalized 450k data to WGBS data, Wang et al. found that PBC and Quantile normalization plus BMIQ normalization (QN.BMIQ) performed better than other normalization methods [24]. Similarly, Wu et al. [13] found BMIQ to outperform other normalization methods, and Marabita et al. [25] found BMIQ and QN.BMIQ to be the most effective normalization methods for correcting probe-type bias. Yet, some researchers suggest that background correction or other forms of normalization are unnecessary as they may introduce a new source of variance [13, 26]. Unfortunately, very few studies have compared available normalization methods using the new EPIC array.

Replication studies using Illumina BeadChips (i.e. 450k or EPIC) have shown that at the array level, DNA methylation values are highly correlated [6, 14, 27–29]. At the individual probe level, however, thousands of individual CpG sites have been identified as unreliable. Probe reliability is influenced by both biological variation and technical variation [14]. Studies often identify unreliable probes using intraclass correlation coefficients (ICCs) [3, 14, 30, 31]. When using ICC to measure individual CpG sites, many studies find that the majority of CpG sites have low correlation [14, 28, 31]. The low correlation observed amongst the majority of CpG sites largely occurs due to low variation in methylation status, as most CpG sites are usually completely methylated or completely unmethylated [28]. Some researchers have suggested that CpG sites with low ICCs should be excluded from analyses or interpreted with extra caution [30]. However, excluding all of the sites with low variability may lead to the exclusion of important regulatory regions [14].

This study looks to evaluate the reliability of the EPIC array and identify the best normalization techniques using technical replicate samples (e.g. samples characterized twice using the EPIC array) of elderly individuals from Brazil. We also carried out correlation and ICC analyses to explore the extent to which the best normalization approach improved probe reliability and replicability with respect to the raw data.

## Materials and methods

### Study participants and samples

The whole blood samples used in this paper were obtained from the Health, Well-being and Aging (Saúde, Bem-estar e Envelhecimento, SABE) study cohort. SABE is a cohort of census-withdrawn elderly from the city of São Paulo, Brazil, followed-up every five years since the year 2000, with DNA first collected in 2010, and previously described in genomic studies [32, 33]. Samples from 24 elderly adults were collected at two time points for a total of 48 samples. The first time point is the 2010 collection wave, performed from 2010 to 2012, and the second time point was set in 2020 in a COVID-19 monitoring project (9 ± 0.71 years apart). The 24 individuals were 67.41 ± 5.52 years of age (mean ± standard deviation) at time point one, and 76.41 ± 6.17 at time point two and comprised 13 men and 11 women. Individuals are admixed (mean ancestry proportions of 0.65 European, 0.21 African and 0.14 Native American), based on previous global ancestry analyses [33].

### Blood collection and processing

Genomic DNA was extracted from whole peripheral blood samples collected in EDTA tubes. DNA extraction and purification followed manufacturer’s recommended protocols, using Qiagen AutoPure LS kit with Gentra automated extraction (first time point) or manual extraction (second time point), due to discontinuation of the equipment but using the same commercial reagents. DNA was quantified using Nanodrop spectrometer and diluted to 50 ng/uL. To assess the reproducibility of the EPIC array, a subset of 16 of the 48 samples was randomly selected for technical replicates, for a total of 64 samples submitted for further analyses. Whole-genome sequencing data are also available for the samples described above.

### Characterization of DNA methylation using the EPIC array

Approximately 1000 ng of human genomic DNA was used for bisulphite conversion. Methylation status was evaluated using the MethylationEPIC array at The Centre for Applied Genomics (TCAG, Hospital for Sick Children, Toronto, Ontario, Canada), following protocols recommended by Illumina (San Diego, California, USA).

### Processing and analysis of DNA methylation data

The R/Bioconductor packages *Meffil* (version 1.1.0), *RnBeads* (version 2.6.0), *minfi* (version 1.34.0), and *wateRmelon* (version 1.32.0) were used to import, process, and perform quality control (QC) analyses on the methylation data. Starting with the 64 samples, we first used *Meffil* to infer the sex of the 64 samples and compared the inferred sex to reported sex. Utilizing the 59 SNP probes that are available as part of the EPIC array, we calculated concordance between the methylation intensities of the samples and the corresponding genotype calls extracted from their WGS data. We then performed comprehensive sample-level and probe-level QC using the *RnBeads* QC pipeline. Specifically, we (1) removed probes if their target sequences overlap with a SNP at any base, (2) removed known cross-reactive probes (3) used the iterative Greedycut algorithm to filter out samples and probes, using a detection *p* value threshold of 0.01, and (4) removed probes if more than 5% of the samples had a missing value. Since *RnBeads* does not have a function to perform probe filtering based on bead number, we used the *wateRmelon* package to extract bead numbers from the IDAT files and calculated the proportion of samples with a bead number < 3. Probes with more than 5% of samples having a low bead number (< 3) were removed. For the comparison of normalization methods, we also computed detection *p* values using out-of-band probes empirical distribution with the pOOBAH() function in the *SeSAMe *(version 1.14.2) R package, with a *p* value threshold of 0.05, and the combine.neg parameter set to TRUE. In the scenario, where pOOBAH filtering was carried out, it was done in parallel with the previously mentioned QC steps, and the resulting probes flagged in both analyses were combined and removed from the data.

### Normalization methods evaluated

**Fig. 1:**
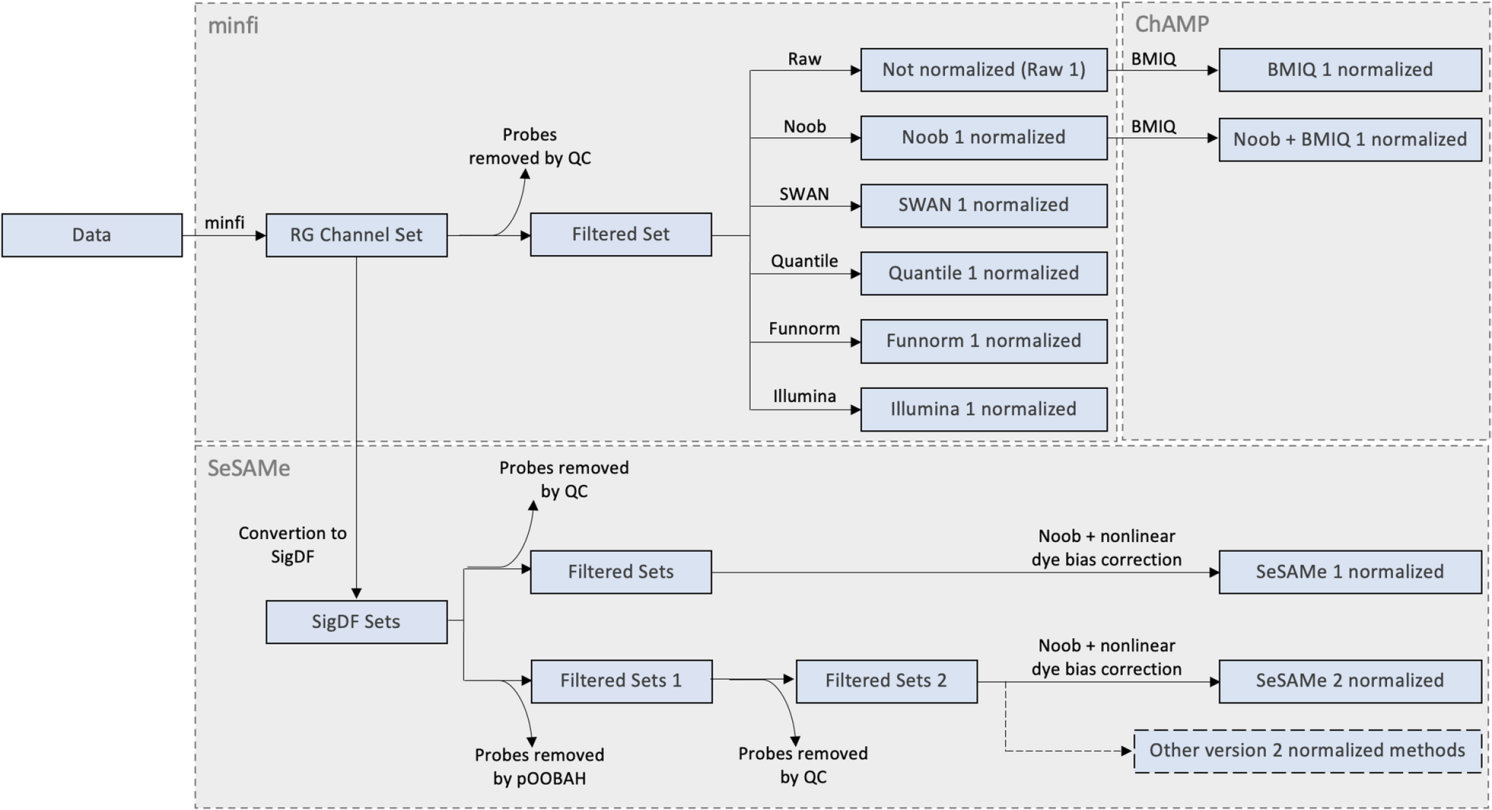
The normalization pipelines for all normalization methods considered

The normalization methods compared in this study were implemented using different R/Bioconductor packages and are summarized in Fig. 1. All data were read into R workspace as RG Channel Sets using *minfi’s* read.metharray.exp() function. One sample that was flagged during QC was removed, and further normalization steps were carried out in the remaining set of 63 samples. Prior to all normalizations with *minfi*, probes that did not pass QC were removed. Noob, SWAN, Quantile, Funnorm, and Illumina normalizations were implemented using *minfi*. BMIQ normalization was implemented with *ChAMP* (version 2.26.0), using Raw input data produced by *minfi’s* preprocessRaw() function. In the combination of Noob with BMIQ (Noob + BMIQ), BMIQ normalization was carried out using as input *minfi’s* Noob normalized data. Noob normalization was also implemented with *SeSAMe*, using a nonlinear dye-bias correction. For all normalization methods, two scenarios were tested. In the first, which we call version 1 (e.g. BMIQ 1, SeSAMe 1, SWAN 1, etc.), *SeSAMe’s* pOOBAH masking was not executed, and the only probes filtered out of the dataset prior to normalization were the ones that did not pass QC in the previous analyses. In the second scenario, which we call version 2 (e.g. BMIQ 2, SeSAMe 2, SWAN 2, etc.), pOOBAH masking was carried out in the unfiltered dataset, and masked probes were removed. This removal was followed by a further removal of probes that did not pass previous QC and that had not been removed by pOOBAH. Therefore, the version 2 of each method had two rounds of probe removal. Methods were then compared by subsetting the 16 replicated samples and evaluating the effects that the different normalization methods had in the beta-value distributions and in the absolute difference of beta values (|*β*|) between replicated samples. Results were plotted either with base R, or with the ggplot2 (version 3.3.6) or limma (version 3.52.2) R packages.

### Reproducibility analyses

We used R (version 4.2.0) and Bioconductor packages to compute summary statistics and conduct statistical analyses. We first examined the distributions of the raw and normalized *β* values, including standard deviation (SD), mean, median, and range (maximum *β*–minimum *β*). To assess the reproducibility and reliability of probe measures, we used the cor() function to compute Pearson’s correlation of the technical replicates at each probe. We then calculated the intraclass correlation coefficients (ICCs) using the icc() function from the *irr* package (version 0.84.1). Since there are many forms of ICCs, we followed the recommendations by Koo and Li [34] and calculated the ICCs under the ‘two-way random effects, absolute agreement, single rater/measurement’ model (with the ‘twoway’, ‘agreement’, and ‘single’ options in the icc() function). Through boxplots and Hexbin scatterplots, we examined the relationship between probe reproducibility, as measured by Pearson’s correlations and ICCs, and the distributions of *β* values (SD and mean).

## Results

### Normalization results

In the current paper, we evaluated probe replicability using raw and normalized beta values. The normalization techniques employed reduce technical variability. The normalization methods considered, and the pipelines used, can be found in Fig. 1. Each normalization method included a quality control step, which filtered out 182,562 probes. Additionally, 17,789 unique probes were filtered using pOOBAH for the second version of each normalization method (e.g. SeSAMe 2). Therefore, the first iteration of the datasets consider 684,274 probes for analyses, whereas the second iterations consider 666,485 probes. Our first set of analyses compare the performance of the first version of each normalization method with the full SeSAMe pipeline, which includes pOOBAH masking (i.e. SeSAMe 2).

Table 1 provides details of the raw and normalized absolute beta value differences (|∆*β*|) for all replicate samples, highlighting the percentage of CpGs surpassing |∆*β*|= 0.05 or 0.1. Additional file 1: Figs. S1 and S2 show these distributions in graphical format. Additional file 1: Fig. S3 depicts the distributions for the individual replicate pairs. With respect to absolute beta-value differences, SeSAMe 2 produced the most favourable results, exhibiting the smallest median |∆*β*| value (0.00815) and the lowest proportion of probes with |∆*β*| > 0.05 and |∆*β*| > 0.10 (1.97% and 0.10%, respectively, Table 1). It is important to note, however, that SeSAMe 2 includes an additional round of probe removal. If we compare only the other methods, which underwent the same probe removal procedure, the best method is either SeSAMe 1 or Noob + BMIQ 1, depending on the criteria used. Noob + BMIQ 1 showed smaller |∆*β*| values for the median and Q1, while SeSAMe 1 showed better results for more extreme values. The additional probe removal using pOOBAH, therefore, seems to have removed a good proportion of poorly replicated probes in SeSAMe 1. Overall, the raw data produced the least favourable results, with the highest median values (0.01627) and the highest proportion of probes with |∆*β*| > 0.05. Quantile 1 and BMIQ 1 normalization produced the highest proportions of CpGs with an |∆*β*| > 0.10 (0.43% and 0.33%).

**Table 1:** Absolute beta-value (|∆*β*|) differences for all replicate samples

	Lower Whisker	Q1	Q2 (Median)	Q3	Upper Whisker	|Δ*β*| > 0.05 (%)	|Δ*β*| > 0.10 (%)	Type	
Raw 1	0.00000	0.00734	0.01627	0.02931	0.06226	6.50	0.30	–	–
BMIQ1	0.00000	0.00578	0.01348	0.02627	0.05699	5.74	0.33	Quantile	Within-array
SWAN 1	0.00000	0.00588	0.01286	0.02357	0.05009	4.27	0.30	Quantile	Within-array
Illumina 1	0.00000	0.00554	0.01206	0.02167	0.04587	2.86	0.21	Correction*	Within-array
Quantile 1	0.00000	0.00523	0.01164	0.02148	0.04587	3.63	0.43	Quantile	Between-array
Noob 1	0.00000	0.00379	0.00911	0.01822	0.03987	2.47	0.21	Correction*	Within-array
Funnorm 1	0.00000	0.00362	0.00890	0.01792	0.03936	2.67	0.30	Mixed	Between-array
SeSAMe 1	0.00000	0.00342	0.00840	0.01763	0.03895	2.49	0.21	Correction*	Within-array
Noob + BMIQ 1	0.00000	0.00333	0.00829	0.01771	0.03928	2.75	0.25	Mixed	Within-array
SeSAMe 2	0.00000	0.00333	0.00815	0.01709	0.03773	1.97	0.10	Correction*	Within-array

*Correction: includes background correction and dye-bias correction

Additional file 1: Fig. S4 shows a table depicting the number of probes with |∆*β*| > 0.10, as well as the number of replication pairs in which the probes exceeded this threshold (1–16). This is also shown in graphical format in the figure. In concordance with the results described above, SeSAMe 2 exhibits the best performance, with a total of 7942 CpGs exceeding |∆*β*| > 0.10 between replicate pairs. Noob normalization was second in terms of performance, with a total of 13,871 probes, followed by SeSAMe 1 with 14,236 probes. Quantile and BMIQ produced the least favourable results with a total of 27,828 and 21,865 CpGs exceeding |∆*β*| > 0.10 between replicate pairs, respectively.

All samples produced an expected bimodal beta-value distribution, with most probes having beta values close to either 0 or 1 (See Additional file 1: Fig. S5 for density plots). However, BMIQ diverges slightly from the expected shape, as it does not produce a second larger peak. For the data normalized with BMIQ, the two peaks are approximately the same height on the graph.

**Fig. 2:**
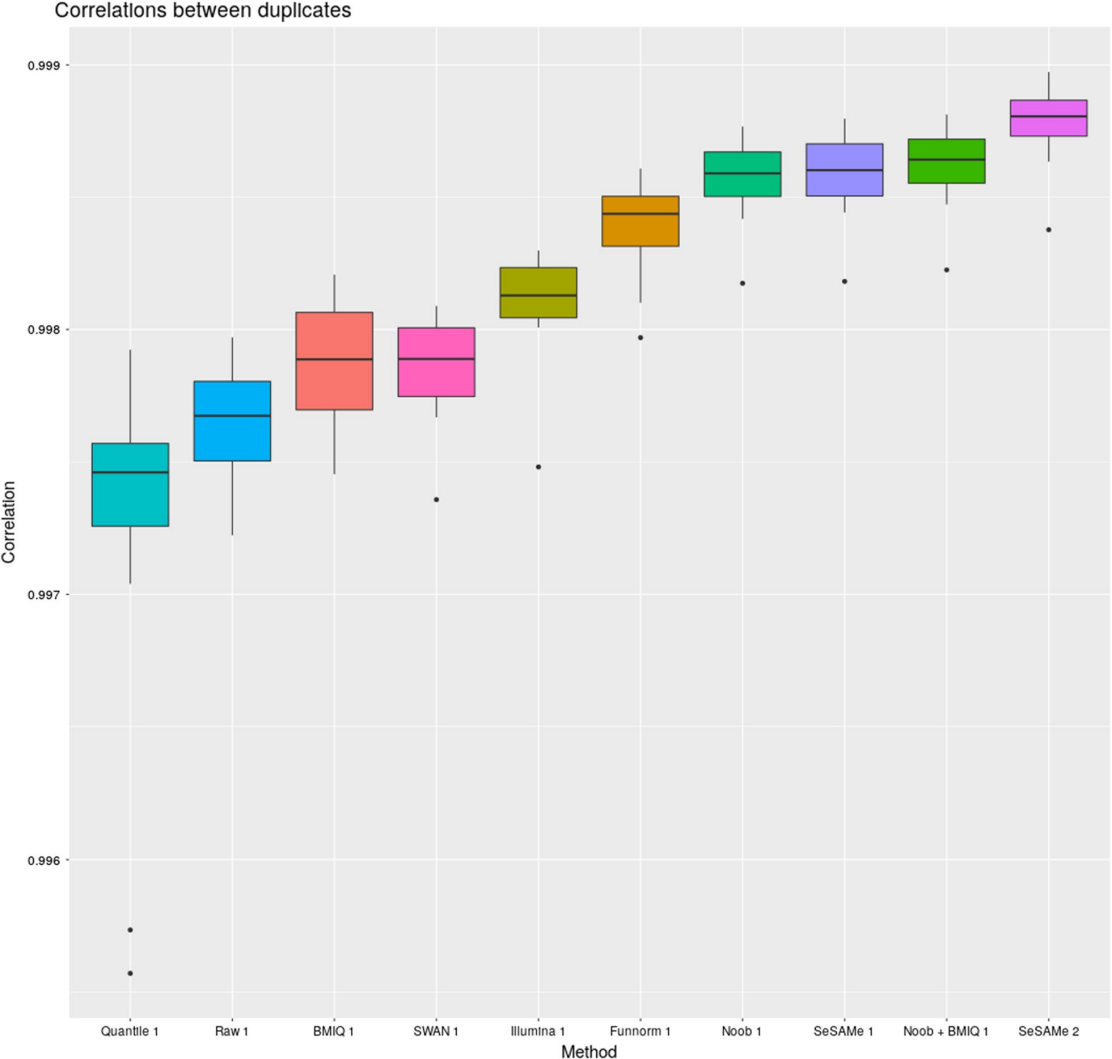
Boxplot of correlation between the replicate samples for each method, ordered by median

Figure 2 shows a Boxplot of correlation between the replicate samples for each method, ordered by median. All datasets exhibited high correlations (> 0.997). However, the highest correlations were observed for SeSAMe 2, followed by Noob + BMIQ 1, SeSAMe 1, and Noob 1. The lowest correlations were observed for Quantile normalization, followed by the raw data.

Due to the superior performance of SeSAMe 2 after pOOBAH probe filtering, available in the SeSAMe pipeline, we conducted a second set of analyses, applying the pOOBAH filtering to the raw data and other normalization methods (e.g. BMIQ 2, SWAN 2, Noob 2). SeSAMe 2 continued to be the best performing method when considering the proportion of probes with |∆*β*| > 0.05 and correlation between the replicate samples (See Additional file 1: Table S1, Figs. S6 and S7). However, when considering the number of probes with |∆*β*| > 0.10, SeSAMe 2 was outperformed by Illumina 2 normalization (0.10% and 0.09%, respectively, Additional file 1: Table S1). Overall, SeSAMe 2 remained the best performing normalization method, though filtering with pOOBAH did substantially increase the performance of the raw data and other normalization methods.

### Pearson’s correlation and intraclass correlation coefficient results

Pearson’s correlation and intraclass correlation coefficient (ICC) results were generated using raw beta values as well as beta values normalized using the SeSAMe 2 normalization method. SeSAMe 2 normalization was used for these detailed analyses as it was deemed the most effective normalization method in this study based on the metrics discussed in the previous section. Correlational methods were performed on the *n* = 32 samples (16 replicate pairs) for *n* = 666,485 probes remaining after quality control filtering.

**Fig. 3:**
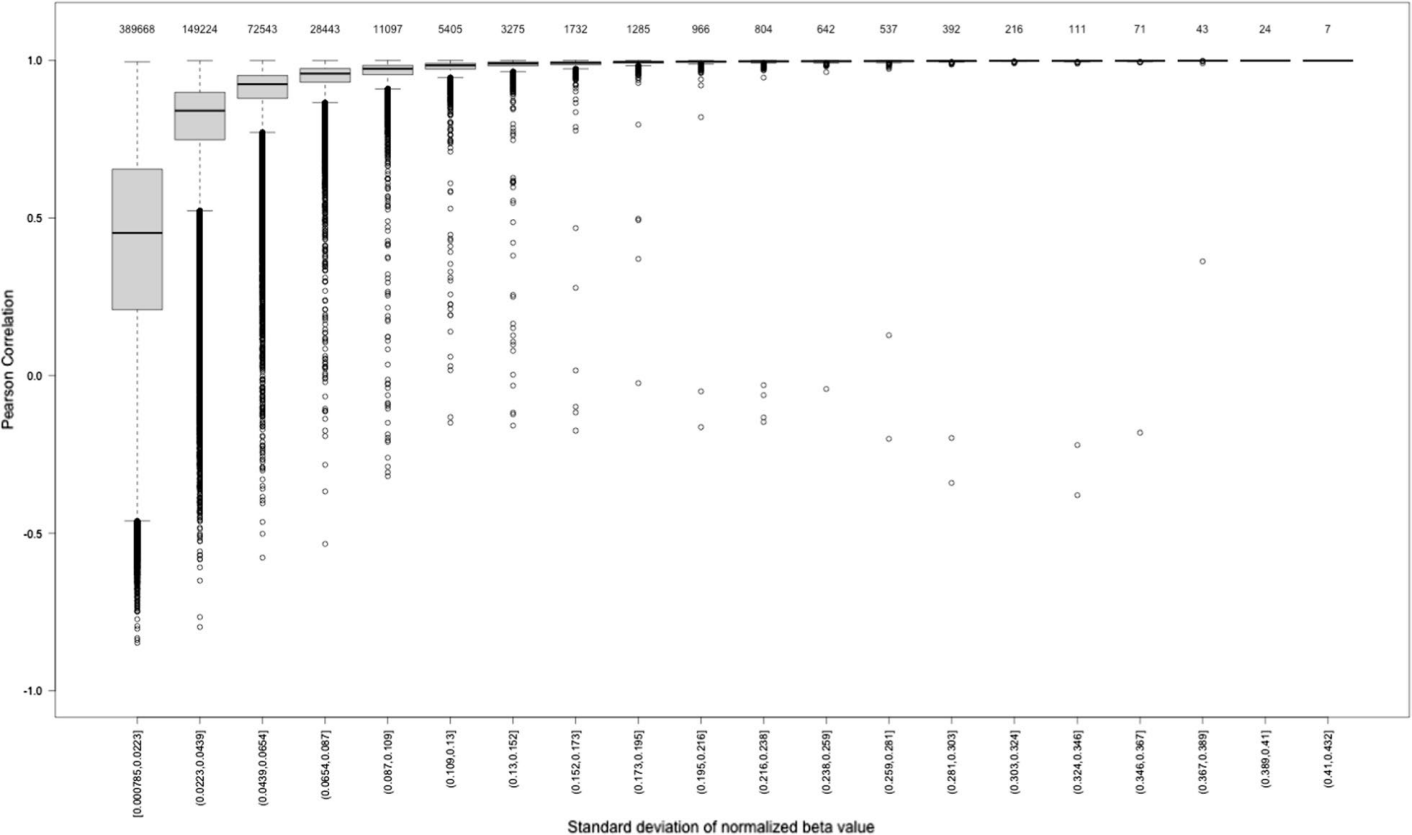
Box plot showing SD versus Pearson’s correlation based on SeSAMe 2 normalized beta values, using equidistant SD categories in the *X* axis

The whole-array Pearson’s correlation of beta values for each of the 16 replicates pairs was high using both raw and normalized data (Avg. *R*^2^ > 0.9975; See Table 2). Pearson’s correlations were also estimated for individual probes. When considering individual probes, the correlation was much lower, with a mean of 0.5165 and median of 0.5840 for raw data and a mean of 0.5979 and a median of 0.6853 for normalized data (See Table 3). We explored in detail the distribution of Pearson’s correlation values for raw and SeSAMe 2 beta values across mean beta categories (Hexbin, equidistant categories, and percentiles) and across standard deviation of beta categories (Hexbin, equidistant categories, and percentiles). These are shown in Additional file 1: Figs. S8–S13. Pearson’s correlation values are substantially lower when mean beta values are very low or very high, or when the standard deviations are low (e.g. there is very low variation in beta values across the samples). It is important to note that most probes have very low standard deviations. As an example, Fig. 3 shows a box plot of Pearson’s correlation by equidistant SD categories of beta values normalized by SeSAMe 2. The majority of the probes (389,668 or 58.5%) are included in the first category (SD < 0.0223) and show low Pearson’s correlation values (mean < 0.5), whereas only 1.5% of the probes (10,105) are included in the top 14 categories (e.g. SD > 0.13), which show high Pearson’s correlation values. Table 3 highlights that there is an improvement in Pearson’s correlation values when using SeSAMe 2 normalized beta values with respect to the raw beta values. Applying nonparametric correlation methods (Spearman’s rank correlation) produced similar results to those observed using Pearson’s correlation.

**Table 2:** Pearson’s correlation for each pair of replicate samples across all probes for raw and normalized data

Data type	Minimum	1st Quantile	Median	Mean	3rd Quantile	Maximum
Raw beta values	0.9975	0.9978	0.9979	0.9979	0.9980	0.9982
SeSAMe 2 normalized beta values	0.9984	0.9987	0.9988	0.9988	0.9989	0.9990

**Table 3:** Pearson’s correlation observed for individual probes for raw and normalized data

Data type	Minimum	1st Quantile	Median	Mean	3rd Quantile	Maximum
Raw beta values	− 0.8051	0.2473	0.5840	0.5165	0.8408	0.9999
SeSAMe 2 normalized beta values	− 0.8477	0.3769	0.6853	0.5979	0.8807	1.0000

To assess the reliability of individual probes, ICC analysis was performed. Probes with an ICC > 0.50 are considered to have acceptable reliability, while probes with an ICC value < 0.50 were deemed as unreliable [14]. We first used raw methylation data from the 16 pairs of replicates to calculate the ICC for each CpG site on the EPIC array and then calculated the ICC for the normalized data. Using the raw data, 301,103 of the 666,485 probes (45.18%) have an ICC > 0.50, 151,647 (22.75%) have an ICC > 0.75, and 53,262 (7.99%) have an ICC > 0.90. After SeSAMe 2 normalization, 408,917 (61.35%) probes have an ICC > 0.50, 256,710 (38.52%) have an ICC > 0.75, and 117,470 (17.63%) have an ICC > 0.90. Therefore, normalizing the data using SeSAMe 2 substantially increased the number of probes with an ICC > 0.50. Table 4 provides summary details about the distribution of ICC values for raw and normalized data. The mean and median beta values after normalization are higher than the raw values (Table 5). Similarly to what was done for the correlation analyses, we explored in detail the distribution of ICC values for raw and SeSAMe 2 values based on mean beta values and the standard deviation of beta values, and these results are summarized in graphical format in Additional file 1: Figs. S14–S19. In agreement with our Pearson’s correlation analysis, ICC values are substantially lower when mean beta values that are low or high, or when standard deviations of beta values are low.

**Table 4:** Intraclass correlation results for raw and normalized data

Data type	Minimum	1st Quantile	Median	Mean	3rd Quantile	Maximum
Raw	− 0.8903	0.1886	0.4461	0.4385	0.7264	0.9999
SeSAMe 2 normalized	− 0.9248	0.3247	0.6327	0.5656	0.8553	0.10000

**Table 5:** Mean beta values of raw and normalized data

Data type	Minimum	1st Quantile	Median	Mean	3rd Quantile	Maximum
Raw	0.006653	0.209338	0.727574	0.573223	0.852141	0.990649
SeSAMe 2 normalized	0.01109	0.24068	0.83598	0.63554	0.93684	0.99003

## Discussion

In this study, we used 16 replicate pairs to systematically assess and compare the performance of raw (unnormalized) data and various normalization techniques (i.e. BMIQ, Quantile, Funnorm, SWAN, Illumina, Noob, Noob + BMIQ, SeSAMe) using EPIC array methylation data. After normalization, we used three metrics to evaluate the reproducibility of the 16 technical replicates: absolute beta-value difference (|∆*β*|), overlap of non-replicated CpGs between replicate pairs, and effect on beta-value distributions. We also used Pearson’s correlation and intraclass correlation coefficient (ICC) to evaluate whole-array and probe-level reliability and reproducibility.

### Normalization method performance

Our results indicate that quantile-based normalization methods (i.e. Quantile, BMIQ, and SWAN), especially BMIQ, had overall worse performance compared to the other normalization methods when considering absolute beta-value difference and overlap of non-replicated CpGs between replicate pairs. In contrast, normalization methods that utilize Noob normalization such as SeSAMe, Noob, and Noob + BMIQ performed more favourably in all three metrics evaluated. Overall, SeSAMe 2 was the best performing normalization method of the methods evaluated. Much of SeSAMe 2’s superior performance can be attributed to the improved QC carried out by pOOBAH, as can be seen by comparing SeSAMe 1 and 2. Using pOOBAH as an additional QC method also improved the performance of the other normalization methods, indicating that it is a beneficial QC step for EPIC array data. However, even without pOOBAH (SeSAMe 1), SeSAMe showed better results when compared to most other methods, being tied in second with Noob + BMIQ 1, with Noob + BMIQ 1 producing better results for the median and Q1, and SeSAMe 1 producing better results for more extreme values. These results differ from some previous studies that found quantile-based methods, such as BMIQ, to outperform other normalization methods using 450k array data [13, 24, 25], but are supported by more recent studies that find Noob-based normalization methods to have the best performance for Illumina arrays [21, 35, 36].

Quantile normalization and Funnorm were the only between-array normalization methods considered in this study. Between-array normalization reduces array-to-array variation through adjusting measures on a global scale [37]. The issue with Quantile normalization likely has to do with how it normalizes data, in that it forces ‘the empirical marginal distributions of the samples to be the same, which removes all variation in this statistic’ [19], p. 2]. While still a between-array quantile-based method, Funnorm overcomes this issue by only removing variation explained by a set of covariates [19]. Therefore, Funnorm is able to remove covariates that are associated with technical variation, which may be independent of covariates associated with biological variation [19]; this is likely why it performed better than the other quantile-based methods considered in this study.

Even though BMIQ and SWAN are quantile-based normalization methods, different distributions between samples should not be an issue for these methods as they use within-array normalization. Within-array normalization methods involve background correction, dye-bias correction, and type I and II probe scaling [37]. SWAN matches type I and II beta distributions by separately applying Quantile normalization for different subsets of probes [17], while BMIQ attempts to fit the distribution of type II probes to that of type I probes, after applying a three-state (methylated-M, unmethylated-U, and hemimethylated-H) beta-mixture model to type I and II probes [16]. BMIQ was found to be the worst performing normalization method for all three metrics considered, especially when considering extreme values. SWAN also performed poorly when considering absolute beta-value difference (|∆*β*|) and overlap of non-replicated CpGs between replicate pairs, as it produced only marginally better results when compared to the raw data and BMIQ. Dedeurwaerder et al. [37] similarly found that when comparing 450k and BPS data, SWAN did not improve data quality. Additionally, Dedeurwaerder et al. [37] also similarly found that while BMIQ produced a lower boxplot median value than the raw data for absolute difference, it also produced a higher boxplot whisker, indicating that while BMIQ can improve median values, it also performed worse for extreme values (See Additional file 1: Fig. S2). BMIQ not only underperformed when considering absolute beta-value difference (|∆*β*|) and overlap of non-replicated CpGs between replicate pairs, but also diverged from the expected bimodal distribution (See Additional file 1: Fig. S5). Xu et al. [36] similarly found BMIQ to diverge from expectation, as the distributions produced by BMIQ were distorted and discontinuous when evaluated using 450k and EPIC array data. While it is not entirely clear why SWAN and BMIQ performed poorly, the results indicate that quantile-based normalization methods (whether within-array or between-array) were the worst performing normalization methods for our sample.

Few studies have compared the performance of SeSAMe to other available normalization methods; however, recent studies have highlighted that SeSAMe compares favourably to other approaches. For example, Vanderlinden et al. found that SeSAMe outperformed the quantile-based SWAN normalization when harmonizing data from the 450k and EPIC platforms [38]. Similarly, when considering 26 normalization pipelines, which included within-array and between-array normalization methods, Foox et al. found SeSAMe to be one of the best performing normalization methods [39]. The normalization methods considered include no normalization (raw), SWAN, PBC, Regression on Correlated Probes (RCP), Quantile normalization, Funnorm, Enmix, dasen, SeSAMe, and Gaussian Mixture Quantile Normalization (GMQN) [39]. The researchers found Funnorm + RCP to exhibit the best performance when considering which method had more variance explained by cell line across the epigenome, with a median of 90.4% [39, p. 13]. SeSAMe also performed favourably with a median of 90% [39, Fig. 5a]. Therefore, while the results of this study are not directly comparable to that of Vanderlinden et al. and Foox et al. [38, 39], SeSAMe normalization has been found to be a powerful normalization tool.

The overlap in non-replicated CpGs shown in Additional file 1: Fig. S4 shows CpGs that are being consistently poorly replicated (|∆*β*| > 0.10) in many sample pairs. Out of a universe of ~850,000 CpGs, the fact that a few have poor replication in multiple sample pairs indicates that there is some underlying problem with their probes. For example, using Funnorm, 363 probes showed |∆*β*| values higher than 0.1 in 7 or more sample pairs. Even for the best performing approach, SeSAMe 2, there were 78 CpGs that had poor replication in 7 or more sample pairs, pointing to a consistent source of error for these probes. On the other hand, for the majority of CpGs that failed in only one sample pair, there is a higher possibility that these failures were due to random errors.

### Pearson correlation and ICC

Replicate samples were used to assess the EPIC array replicability using both Pearson’s correlations and ICC. As with previous research using Illumina arrays [13, 14, 18, 29, 40], the EPIC array was found to be highly reproducible (*r* > 0.99) at the whole-array level for both raw and normalized data. However, previous studies have found that whole-array correlations are also high between different individuals [14], suggesting that whole-array Pearson’s correlation may be a poor metric for reproducibility studies. Additionally, while the EPIC array was found to be highly reproducible at the whole-array level, it was determined to be less reproducible at the probe level. Probes found to have lower correlations often exhibited low biological variability with mean methylation values being either very low or very high and having small SDs. Lower correlations in probes with low biological variations have similarly been found in studies comparing the 450k and EPIC arrays [28, 41].

The ICC results of this study are in line with previous studies [14, 27, 28, 31], as we found that when considering the raw data, the majority (> 50%) of CpG sites have low correlation. Similarly to the Pearson’s correlation results, CpGs exhibiting low ICC were primarily CpGs that exhibit low variation in methylation, as the majority of probes exhibiting either high or low mean values and smaller SDs have ICC values < 0.50 (See Additional file 1: Figs. S14–S16). Probes that exhibit beta values close to 0 or 1 typically have relatively smaller SDs compared to probes with intermediate beta values. In contrast, few probes with SDs > 0.1 have ICC values < 0.50 (See Additional file 1: Figs. S17–S19). Therefore, probe reliability appears to largely be the result of biological variation rather than technical measurement variation [14]. CpGs with poor ICC reliability have also been found to have lower statistical power and may be more likely to create false-positive findings [42]. Removing probes with low ICC values can decrease false-positives and increase power; however, excluding CpGs with low reliability could lead to the exclusion of important regulatory regions [14].

Normalizing the data with SeSAMe 2 was found to substantially improve ICC values, through increasing the proportion of probes with an ICC value > 0.50 from 45.18% (raw data) to 61.35% (SeSAMe 2). Other pre-processing and normalization methods, such as background correction and dye-bias correction pre-processing methods, have also been found to increase the number of probes with acceptable reliability [14]. However, even with the improvements afforded through data normalization, this study demonstrates that ICC may not be the best correlational method for assessing probe reliability as CpGs with little biological variation are often considered unreliable based on the mean beta value and SD data. Additionally, calculating ICC also requires assaying replicate samples, which adds a greater cost with very little return [14].

## Limitations

The main limitation of this study is the relatively small sample size of *n* = 16 replicate pairs, as it has been previously suggested that 30 replicate samples will provide reasonably good agreement with ICC classification [14]. While the sample is small, the results of this study demonstrate that Noob-based normalization methods may be more suitable for EPIC array normalization and support previous research in demonstrating that CpGs with little biological variation are often considered unreliable when using ICC or probe-level Pearson’s correlations. This study also shows that applying SeSAMe 2 resulted in a substantial increase in the number of probes with acceptable reliability (ICC > 0.5).

## Conclusion

In conclusion, SeSAMe 2 (SeSAMe with pOOBAH masking and additional QC round) was found to be the best performing normalization method based on the metrics evaluated. It is important to note that the number of probes used in the SeSAMe 2 pipeline is lower than the number of probes used for the other normalization approaches (666,485 vs. 684,274 probes), due to the exclusion of approximately 18,000 probes after pOOBAH masking. We repeated the analyses for all the normalization methods excluding the probes identified by pOOBAH masking, and as expected, there is an improvement in the performance of all the methods. These results are provided in Additional file 1: Table S1. After removing these extra probes, SeSAMe 2 remains the top performing method, but other normalization approaches provide very similar results. For example, the median |∆*β*| observed with Noob + BMIQ 2 is the same as that observed with SeSAMe 2 (0.00815), and the proportion of probes with |∆*β*| values higher than 0.05 and 0.1 is very similar for Noob 2 and SeSAMe 2. The improvements observed after pOOBAH masking indicate that this would be a useful step to add in QC protocols prior to probe normalization. In general, our study indicates that noob-based normalization methods such as SeSAMe, Noob, and Noob + BMIQ performed well. In contrast, quantile-based normalization methods (Quantile, BMIQ, and SWAN) were found to exhibit lower performance compared to the noob-based methods. In line with previous studies, the EPIC array was found to be highly reproducible at the whole-array level, but this is not the case at the probe level, with a relatively large number of probes displaying poor reliability (ICC < 0.5). Additionally, in agreement with previous observations, CpGs with low biological variability tend to have low ICC values. Importantly, normalizing the data with SeSAMe 2 substantially improved ICC estimates, with the proportion of probes with ICC values > 0.50 increasing from 45.18% (raw data) to 61.35% (SeSAMe2). These results emphasize the benefit of using normalization methods when analysing EPIC array data.

## Supplementary Information

**Additional file 1:** Supplementary Figures S1–S19 and Table S1.

## Data Availability

Individual-level datasets analysed in the current study are available from the corresponding author upon reasonable request. Distribution of absolute differences in methylation values (beta values) between replicate samples is characterized with Illumina’s Infinium Methylation EPIC arrays, using different normalization approaches submitted with https://doi.org/10.5061/dryad.cnp5hqc7v.

## Abbreviations

Avg: Average
BMIQ: Beta-mixture quantile normalization
CpG: Cytosine–guanine dinucleotide
Funnorm: Functional normalization
GMQN: Gaussian mixture quantile normalization
ICC: Intraclass correlation coefficient
Noob: Normal-exponential convolution using out-of-band probes
PBC: Peak-based correction
Q1: Quantile 1
QC: Quality control
QN: Quantile normalization
QN.BMQI: Quantile normalization plus beta-mixture quantile normalization
RCP: Regression on correlated probes
SABE: Saúde, Bem-estar e Envelhecimento
SD: Standard deviation
SeSAMe: Sensible step-wise analysis of DNA methylation BeadChips
SNP: Single-nucleotide polymorphism
SSnoob: Single-sample noob
SWAN: Subset-quantiles with microarray normalization
WGBS: Whole-genome bisulphite sequencing

## References

[1] Moore LD, Le T, Fan G (2013). DNA methylation and its basic function. Neuropsychopharmacol.

[2] Smith ZD, Meissner A (2013). DNA methylation: roles inmammalian development. Nat Rev Genet.

[3] Zaimi I, Pei D, Koestler DC, Marsit CJ, De Vivo I, Tworoger SS, Shields AE, Kelsey KL, Michaud DS (2018). Variation in DNA methylation of human blood over a 1-year period using the illumina MethylationEPIC array. Epigenetics.

[4] Brunet A, Berger SL (2014). Epigenetics of aging and aging-related disease. J Gerontol A Biol.

[5] Johnson AA, Akman K, Calimport SR, Wuttke D, Stolzing A, De Magalhaes JP (2012). The role of DNA methylation in aging, rejuvenation, and age-related disease. Rejuvenation Res.

[6] Pidsley R, Zotenko E, Peters TJ, Lawrence MG, Risbridger GP, Molloy P, Djik SV, Muhlhausler B, Stirzaker C, Clark SJ (2016). Critical evaluation of the Illumina MethylationEPIC BeadChip microarray for whole-genome DNA methylation profiling. Genome Biol.

[7] Campagna MP, Xavier A, Lechner-Scott J, Maltby V, Scott RJ, Butzkueven H, Jokubaitis VG, Lea RA (2021). Epigenome-wide association studies: current knowledge, strategies and recommendations. Clin Epigenet.

[8] Teschendorff AE, Relton CL (2018). Statistical and integrative system-level analysis of DNA methylation data. Nat Rev Genet.

[9] Nakabayashi K, Patel VB, Preedy VR (2017). Illumina HumanMethylation BeadChip for genome-wide DNA methylation profiling: advantages and limitations. Handbook of nutrition, diet, and epigenetics.

[10] Wu MC, Kuan PF, Tost J (2018). A guide to Illumina BeadChip data analysis. DNA methylation protocols.

[11] Maksimovic J, Phipson B, Oshlack A (2017). A cross-package Bioconductor workflow for analysing methylation array data. F1000Res.

[12] Wang Z, Wu X, Wang Y (2018). A framework for analyzing DNA methylation data from Illumina Infinium HumanMethylation450 BeadChip. BMC Bioinformatics.

[13] Wu MC, Joubert BR, Kuan PF, Håberg SE, Nystad W, Peddada SD, London SJ (2014). A systematic assessment of normalization approaches for the Infinium 450K methylation platform. Epigenetics.

[14] Xu Z, Taylor JA (2021). Reliability of DNA methylation measures using Illumina methylation BeadChip. Epigenetics.

[15] Bolstad BM, Irizarry RA, Åstrand M, Speed TP (2003). A comparison of normalization methods for high density oligonucleotide array data based on variance and bias. Bioinformatics.

[16] Teschendorff AE, Marabita F, Lechner M, Bartlett T, Tegner J, Gomez-Cabrero D, Beck S (2013). A beta-mixture quantile normalization method for correcting probe design bias in Illumina Infinium 450k DNA methylation data. Bioinformatics.

[17] Maksimovic J, Gordon L, Oshlack A (2012). SWAN: subset-quantile within array normalization for illumina infinium HumanMethylation450 BeadChips. Genome Biol.

[18] Dedeurwaerder S, Defrance M, Calonne E, Denis H, Sotiriou C, Fuks F (2011). Evaluation of the infinium methylation 450K technology. Epigenomics.

[19] Fortin JP, Labbe A, Lemire M, Zanke BW, Hudson TJ, Fertig EJ, Greenwood CMT, Hansen KD (2014). Functional normalization of 450k methylation array data improves replication in large cancer studies. Genome Biol.

[20] Triche TJ, Weisenberger DJ, Van Den Berg D, Laird PW, Siegmund KD (2013). Low-level processing of Illumina Infinium DNA methylation beadarrays. Nucleic Acids Res.

[21] Fortin JP, Triche TJ, Hansen KD (2017). Preprocessing, normalization and integration of the Illumina HumanMethylationEPIC array with minfi. Bioinformatics.

[22] Zhou W, Triche TJ, Laird PW, Shen H (2018). SeSAMe: reducing artifactual detection of DNA methylation by Infinium BeadChips in genomic deletions. Nucleic Acids Res.

[23] van Rooij J, Mandaviya PR, Claringbould A, Felix JF, van Dongen J, Jansen R, Franke L, Consordium B, Hoen PAC, Heijmans B, van Meurs JBJ (2019). Evaluation of commonly used analysis strategies for epigenome-and transcriptome-wide association studies through replication of large-scale population studies. Genome Biol.

[24] Wang T, Guan W, Lin J, Boutaoui N, Canino G, Luo J, Celedón JC, Chen W (2015). A systematic study of normalization methods for Infinium 450K methylation data using whole-genome bisulfite sequencing data. Epigenetics.

[25] Marabita F, Almgren M, Lindholm ME, Ruhrmann S, Fagerström-Billai F, Jagodic M, Sundberg CJ, Ekström TJ, Teschendorff AE, Tegnér J, Gomez-Cabrero D (2013). An evaluation of analysis pipelines for DNA methylation profiling using the Illumina HumanMethylation450 BeadChip platform. Epigenetics.

[26] Pidsley R, Wong CCY, Volta M, Lunnon K, Mill J, Schalkwyk LC (2013). A data-driven approach to preprocessing Illumina 450K methylation array data. BMC Genom.

[27] Dugué PA, English DR, MacInnis RJ, Jung CH, Bassett JK, FitzGerald LM, Wong EM, Joo JE, Hopper JL, Southey MC, Giles GG, Milne RL (2016). Reliability of DNA methylation measures from dried blood spots and mononuclear cells using the HumanMethylation450k BeadArray. Sci Rep.

[28] Logue MW, Smith AK, Wolf EJ, Maniates H, Stone A, Schichman SA, McGlinchey RE, Milberg W, Miller MW (2017). The correlation of methylation levels measured using Illumina 450K and EPIC BeadChips in blood samples. Epigenomics.

[29] Moran S, Arribas C, Esteller M (2016). Validation of a DNA methylation microarray for 850,000 CpG sites of the human genome enriched in enhancer sequences. Epigenomics.

[30] Bose M, Wu C, Pankow JS, Demerath EW, Bressler J, Fornage M, Grove ML, Mosley TH, Hicks C, North K, Kao WH, Zhang Y, Boerwinkle E, Guan W (2014). Evaluation of microarray-based DNA methylation measurement using technical replicates: the atherosclerosis risk in communities (ARIC) study. BMC Bioinf.

[31] Sugden K, Hannon EJ, Arseneault L, Belsky DW, Corcoran DL, Fisher HL, Houts RM, Kandaswamy R, Moffitt TE, Poulton R, Prinz JA, Rasmussen LJH, Williams BS, Wong CCY, Mill J, Caspi A (2020). Patterns of reliability: assessing the reproducibility and integrity of DNA methylation measurement. Patterns.

[32] Naslavsky MS, Yamamoto GL, de Almeida TF, Ezquina SA, Sunaga DY, Pho N, Bozoklian D, Sandberg TO, Brito LA, Lazar M, Bernardo DV (2017). Exomic variants of an elderly cohort of Brazilians in the ABraOM database. Hum Mutat.

[33] Naslavsky MS, Scliar MO, Yamamoto GL, Wang JY, Zverinova S, Karp T, Nunes K, Ceroni JR, de Carvalho DL, da Silva Simões CE, Bozoklian D (2022). Whole-genome sequencing of 1,171 elderly admixed individuals from Brazil. Nat Commun.

[34] Koo TK (2016). Li MYA guideline of selecting and reporting intraclass correlation coefficients for reliability research. J Chiropr Med.

[35] Shiah YJ, Fraser M, Bristow RG, Boutros PC (2017). Comparison of pre-processing methods for Infinium HumanMethylation450 BeadChip array. Bioinformatics.

[36] Xu Z, Niu L, Taylor JA (2021). The ENmix DNA methylation analysis pipeline for Illumina BeadChip and comparisons with seven other preprocessing pipelines. Clin Epigenet.

[37] Dedeurwaerder S, Defrance M, Bizet M, Calonne E, Bontempi G, Fuks F (2013). A comprehensive overview of Infinium HumanMethylation450 data processing. Brief Bioinf.

[38] Vanderlinden LA, Johnson RK, Carry PM, Dong F, DeMeo DL, Yang IV, Norris JM, Kechris K (2021). An effective processing pipeline for harmonizing DNA methylation data from Illumina’s 450K and EPIC platforms for epidemiological studies. BMC Res Notes.

[39] Foox J, Nordlund J, Lalancette C, Gong T, Lacey M, Lent S, Langhorst BW, Ponnaluri VK, Williams L, Padmanabhan KR, Cavalcante R (2021). The SEQC2 epigenomics quality control (EpiQC) study. Genome Biol.

[40] Christiansen SN, Andersen JD, Kampmann ML, Liu J, Andersen MM, Tfelt-Hansen J, Morling N (2022). Reproducibility of the Infinium methylationEPIC BeadChip assay using low DNA amounts. Epigenetics.

[41] Solomon O, MacIsaac J, Quach H, Tindula G, Kobor MS, Huen K, Meaney MJ, Eskenazi B, Barcellos LF, Holland N (2018). Comparison of DNA methylation measured by Illumina 450K and EPIC BeadChips in blood of newborns and 14-year-old children. Epigenetics.

[42] Dugué PA, English DR, MacInnis RJ, Joo JE, Jung CH, Milne RL (2015). The repeatability of DNA methylation measures may also affect the power of epigenome-wide association studies. Int J Epidemiol.

