# Supplementary material for: A systematic evaluation of normalization methods and probe replicability using infinium EPIC methylation data
Source: Clin Epigenetics. 2023 Mar 11;15:41. doi: 10.1186/s13148-023-01459-z (PMC10008016; doi:10.1186/s13148-023-01459-z)
Supplement: Supplementary file 1 — Additional file 1: Supplementary Figures S1–S19 and Table S1. [file 13148_2023_1459_MOESM1_ESM.docx]

**Additional Tables and Figures**

**H. Welsh^1*+^, C. M. P. F. Batalha^2*^, W. Li^3^, K. L. Mpye^1^, N.C. Souza-Pinto^2^, M.S. Naslavsky^4^, E.J. Parra^1^**

^1^ *Department of Anthropology, University of Toronto at Mississauga, Mississauga, Canada*

^2^ *Department of Biochemistry, University of São Paulo, São Paulo, Brazil*

*^3^ The Centre for Applied Genomics, Hospital for Sick Children, Toronto, Canada*

*^4^ Department of Genetics and Evolutionary Biology, University of São Paulo, São Paulo, Brazil*

*^*^These two authors contributed equally to this work.*

*^+^Corresponding author: hayley.welsh@mail.utoronto.ca*


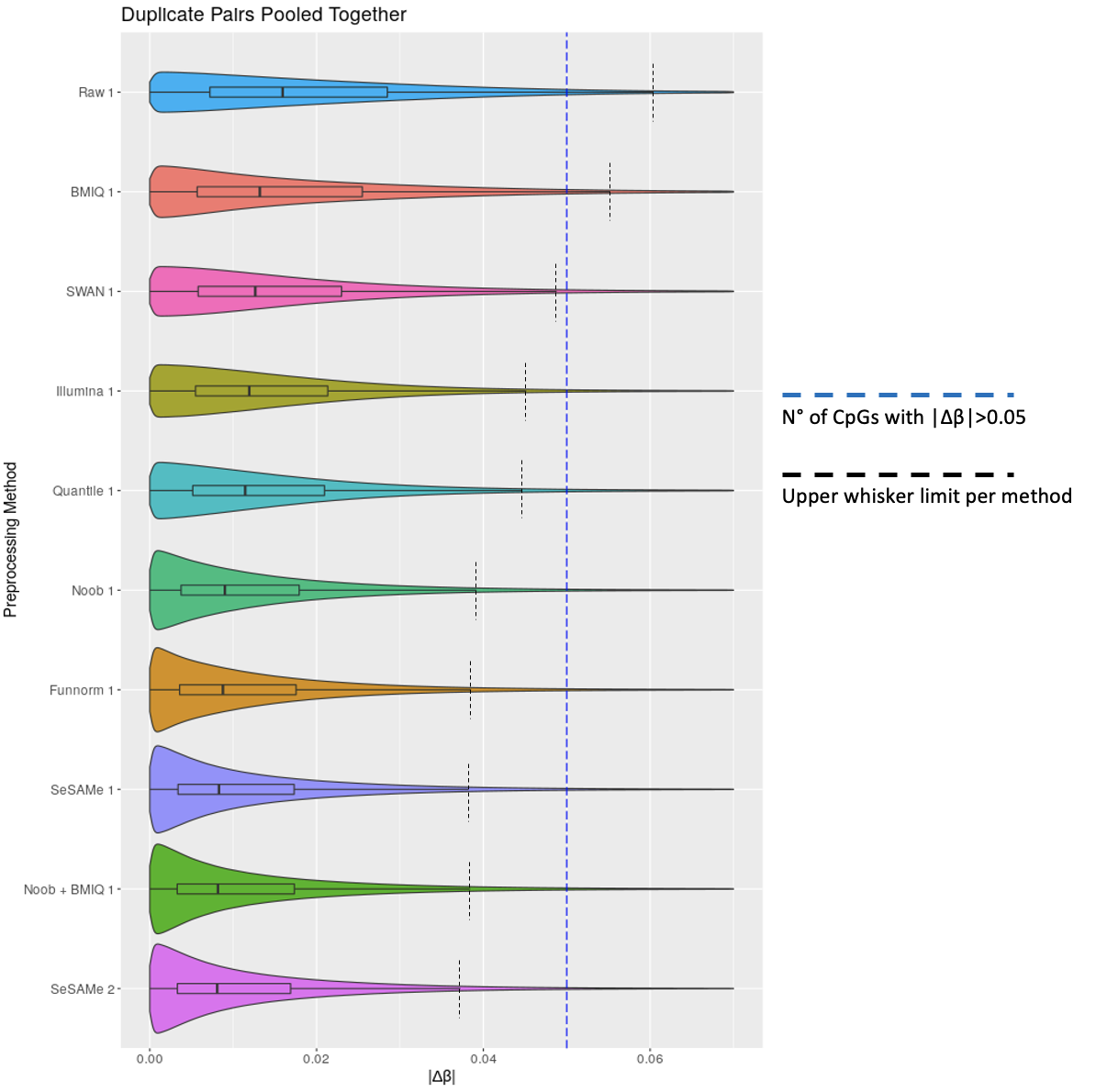


***Figure S1.*** *Violin plot of |∆β|s ordered by median values. All |∆β|s for the 16 replicates were pooled together into one single plot. The plot is broken at 0.07, so more extreme values are not being shown.*

*
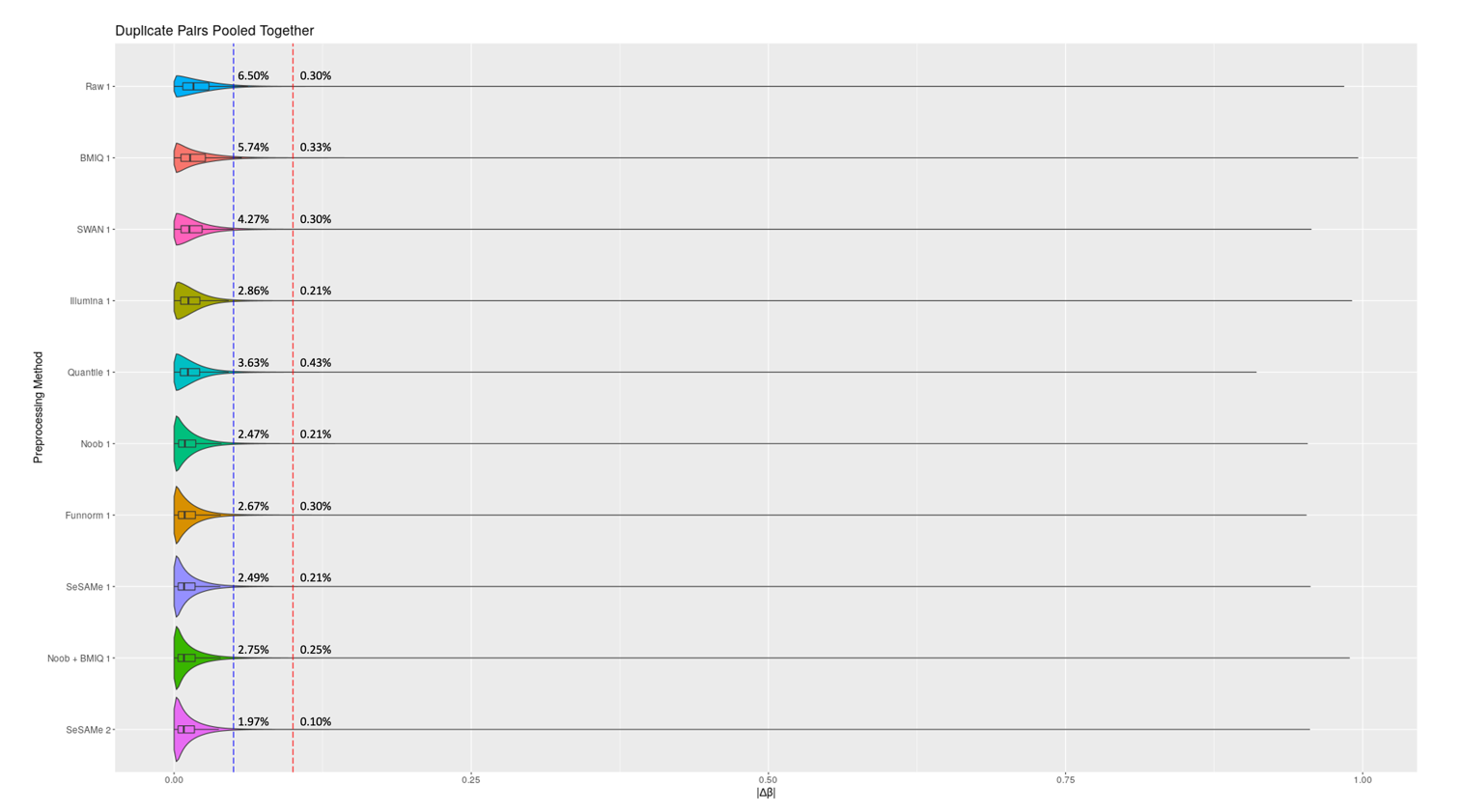
*

***Figure S2.*** *Violin plot of |∆β|s ordered by median values. All |∆β|s for the 16 replicates were pooled together into one single plot. The blue dashed line indicates the percent of CpGs with an |∆β| >0.05 and the red dashed line indicates the percent of CpGs with an |∆β| >0.10.*

*
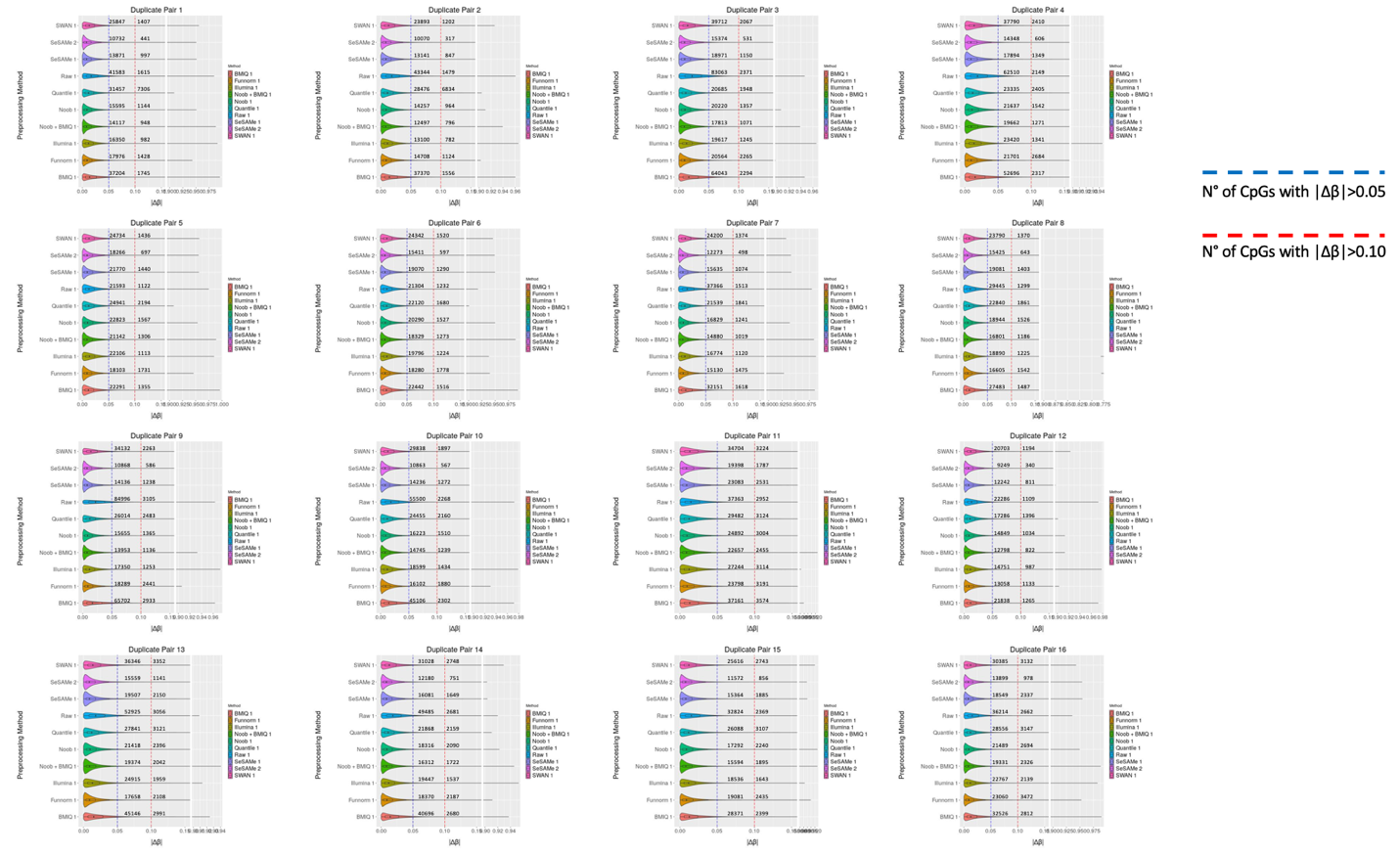
*

***Figure S3.*** *Presents the Violin plot results for absolute difference in beta value for each replicate pair. The blue dashed line indicates the percent of CpGs with an |∆β| >0.05 and the red dashed line indicates the percent of CpGs with an |∆β| >0.10.*

*
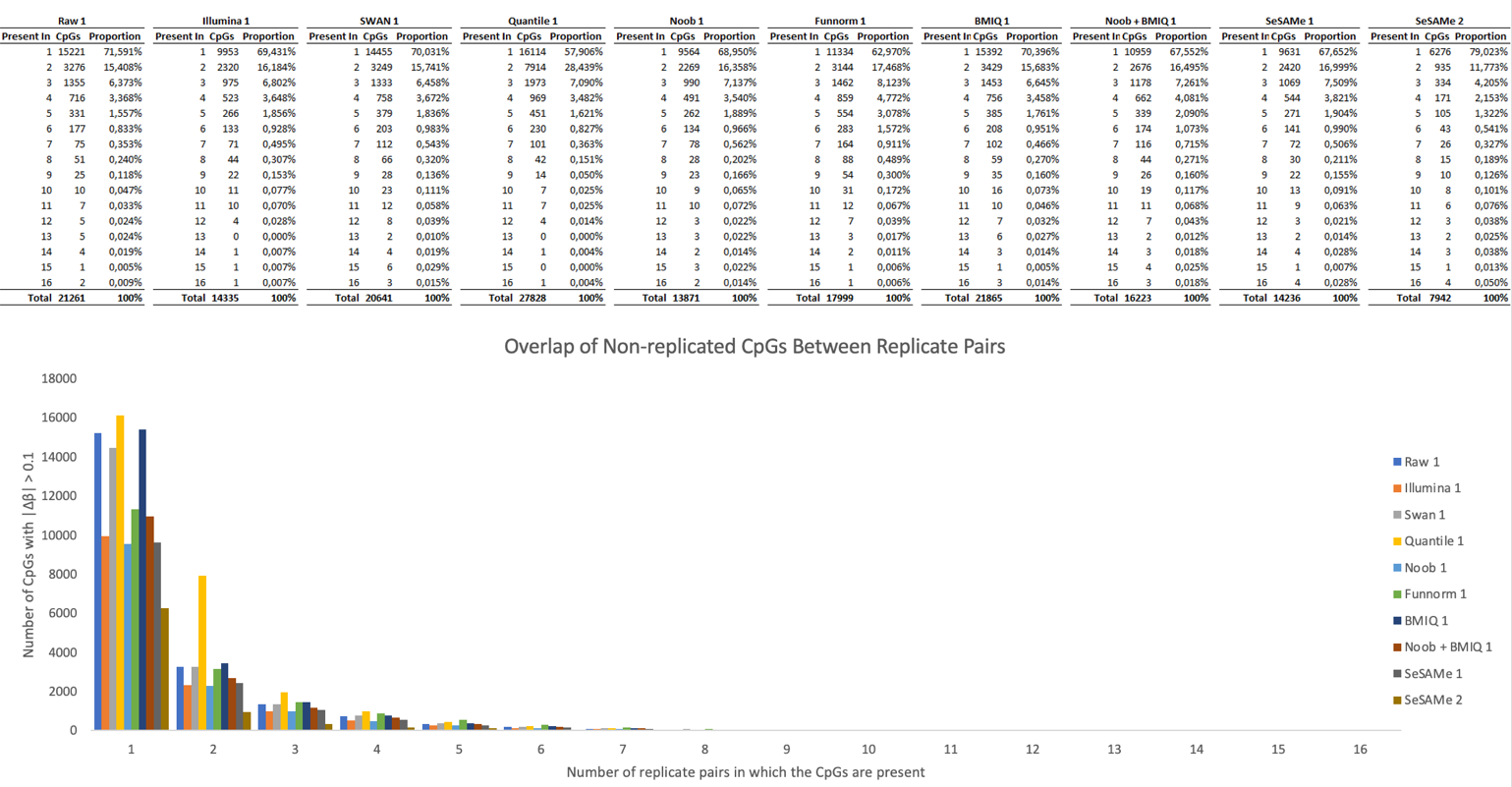
*

***Figure S4.*** *Presents the results for the overlap of non-replicated CpGs (probes with |∆β| >0.10)* *between replicate pairs. SeSAMe 2 exhibits the best performance, with a total of 7,942 CpGs exceeding |∆β| >0.10 between replicate pairs.*

*
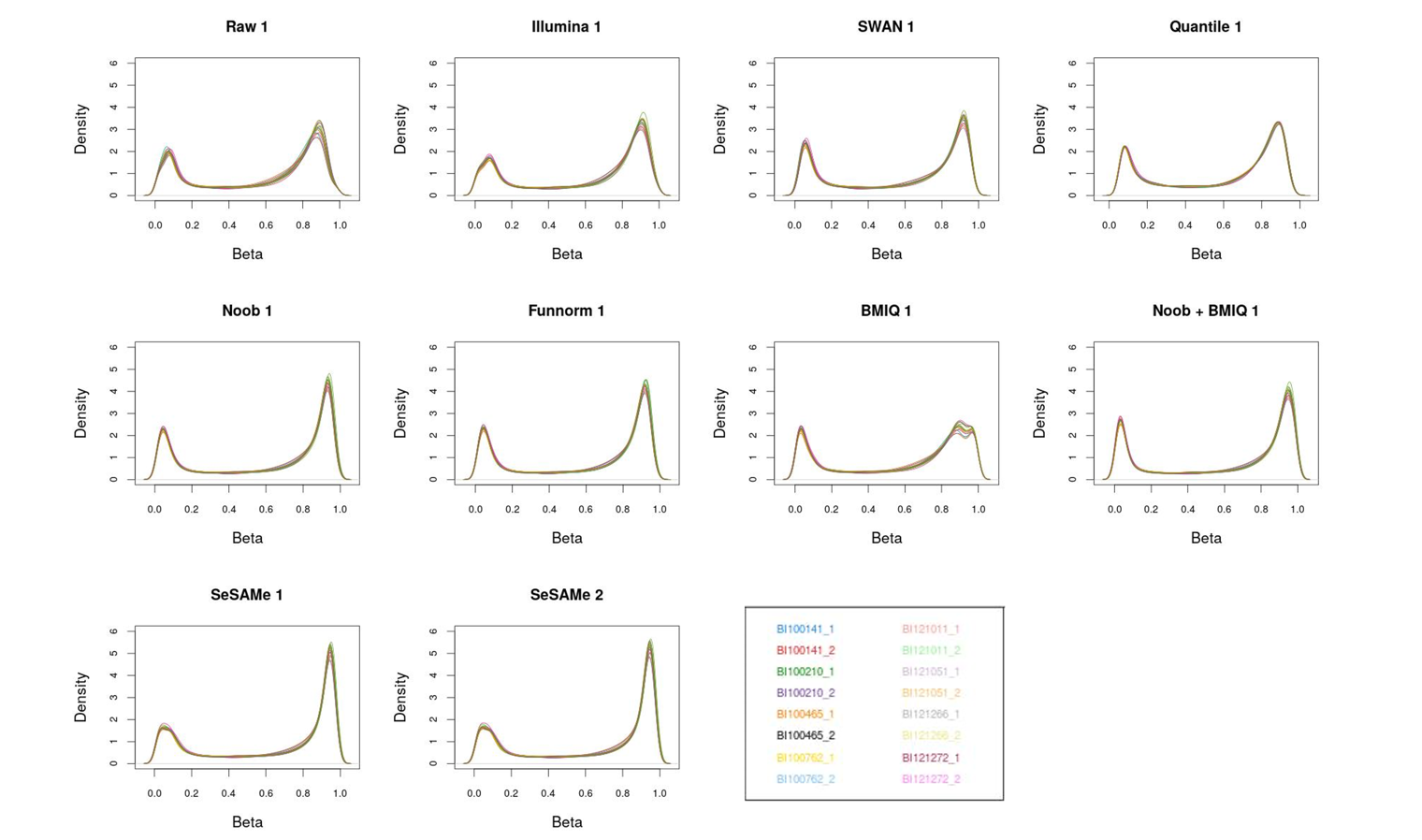
*

***Figure S5.*** *Displays the density plots for the raw and normalized data. The majority of samples produced an expected bimodal beta value distribution, with most probes having beta values close to either 0 or 1, with the exception of BMIQ which diverges slightly from the expected shape.*

Table S1. Absolute beta value (|∆β|) differences for all replicate samples after pOOBAH masking

*
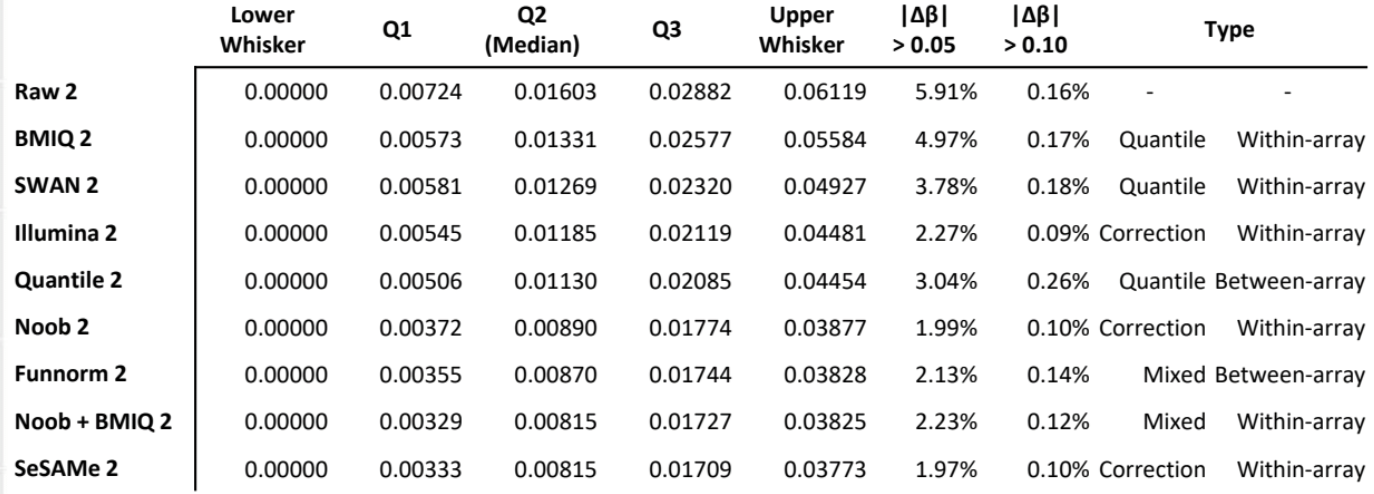
*

*
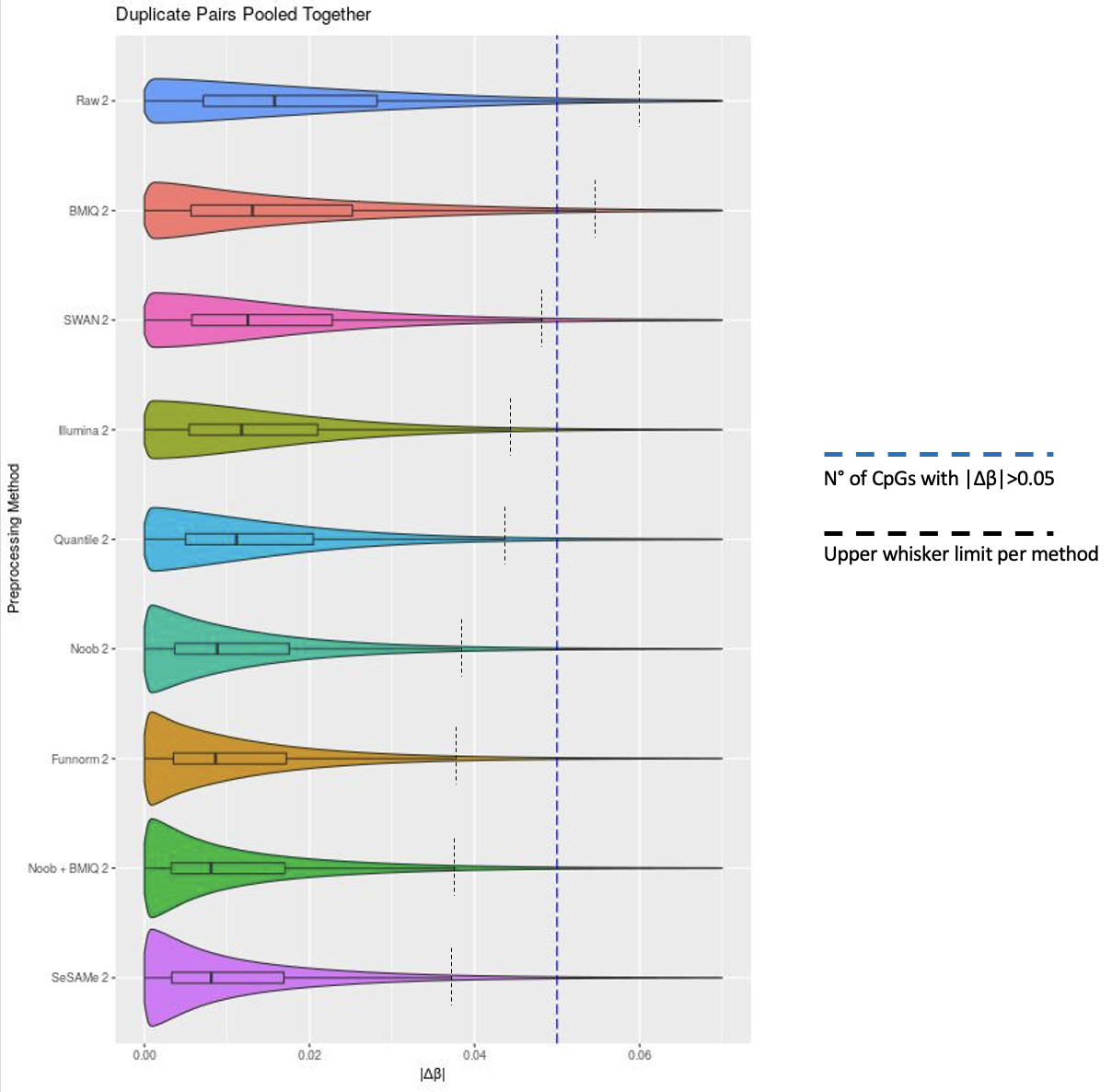
*

***Figure S6.*** *Violin plot of |∆β|s ordered by median values, after pOOBAH masking. All |∆β|s for the 16 replicates were pooled together into one single plot. The plot is broken at 0.07, so more extreme values are not being shown.*

*
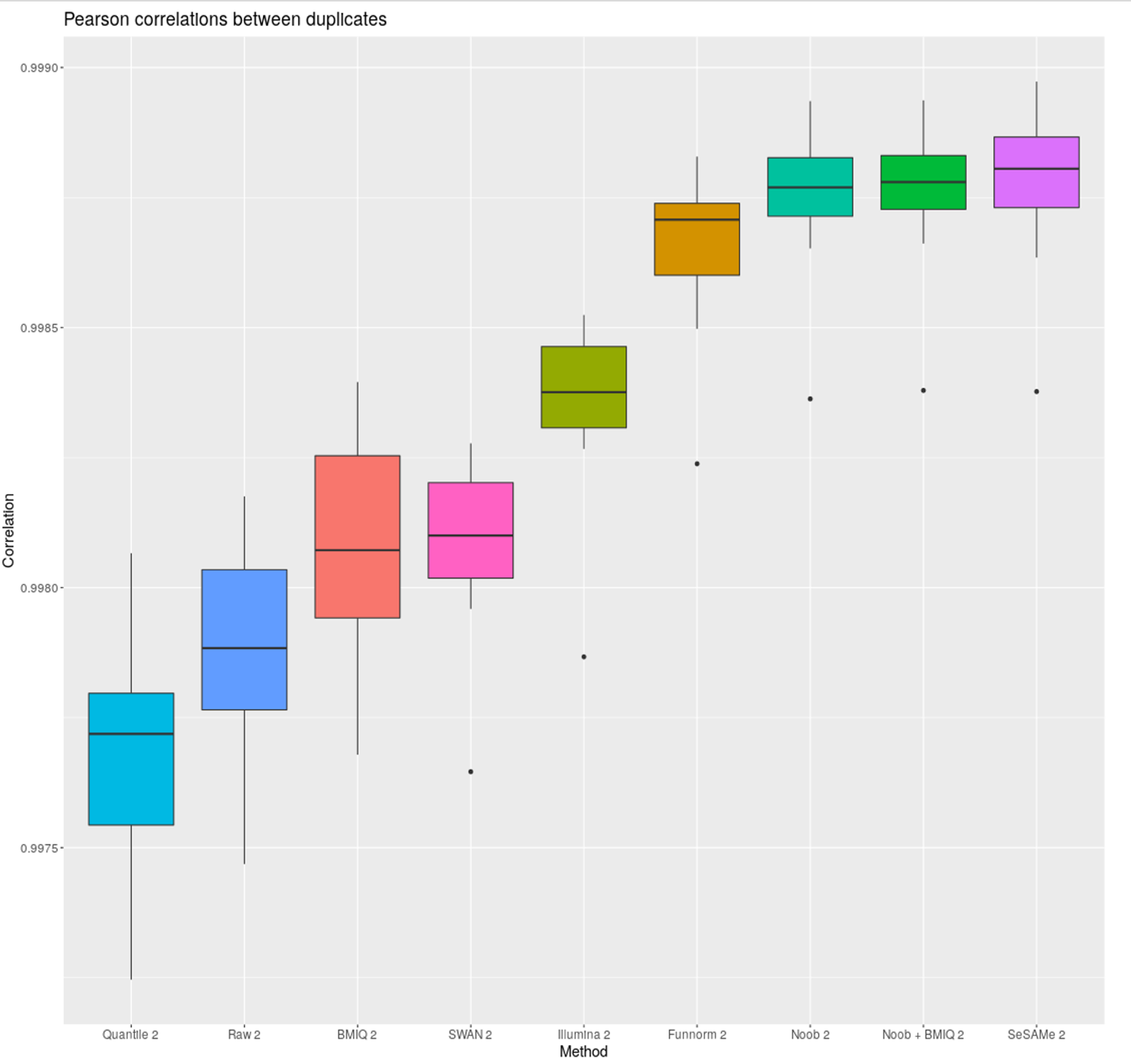
*

***Figure S7.*** *Boxplot of Pearson’s correlation between the replicate samples for each method after pOOBAH masking, ordered by median.*

*
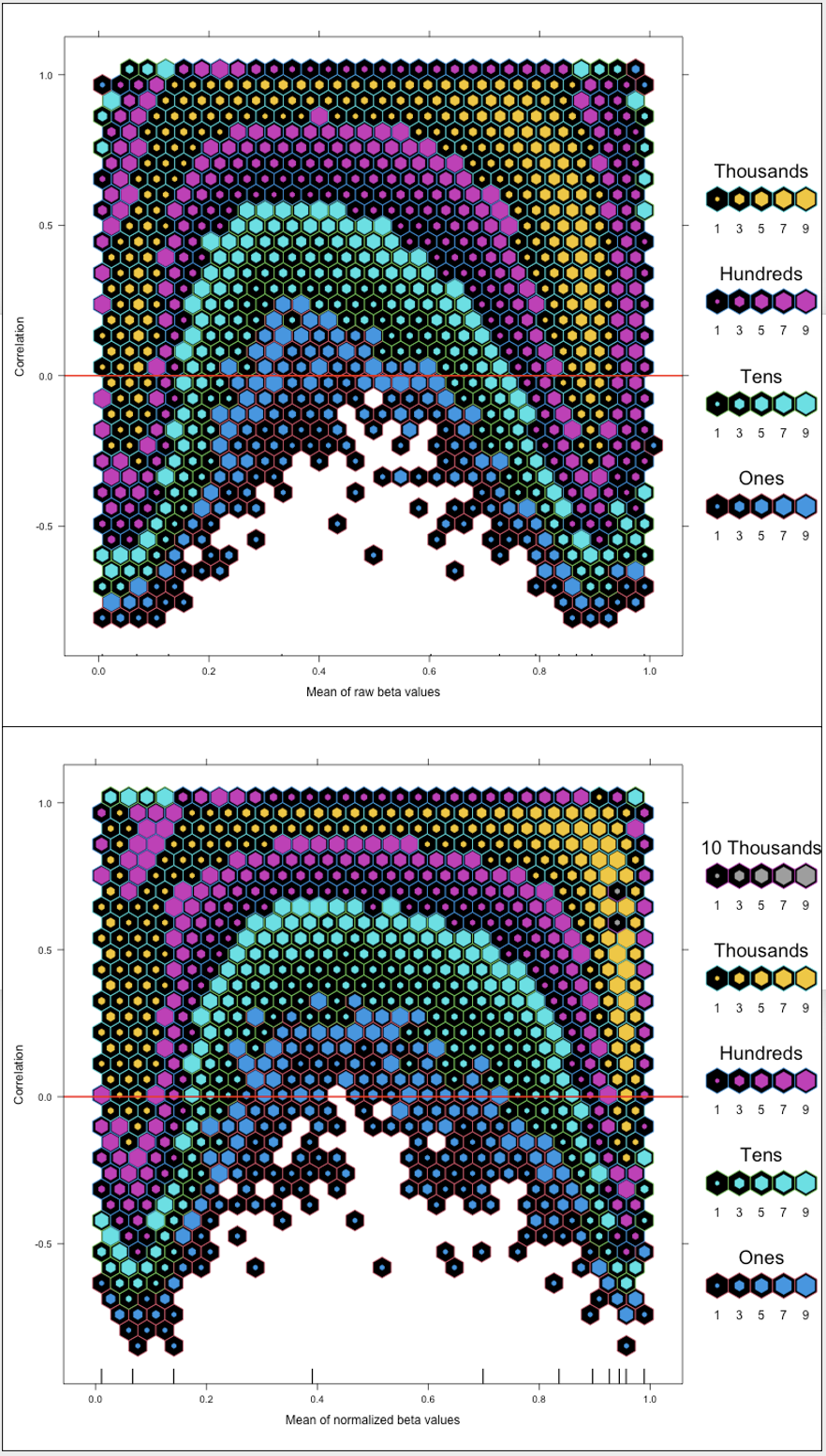
*

***Figure S8.*** *Hexabin scatter plot of mean vs. Pearson’s correlation based on raw beta values (top) and SeSAMe 2 normalized beta values (bottom).* *Pearson’s correlation values are substantially lower when mean beta values are very low or very high (closer to 0 or 1) but improved following normalization with SeSAMe 2.*

*
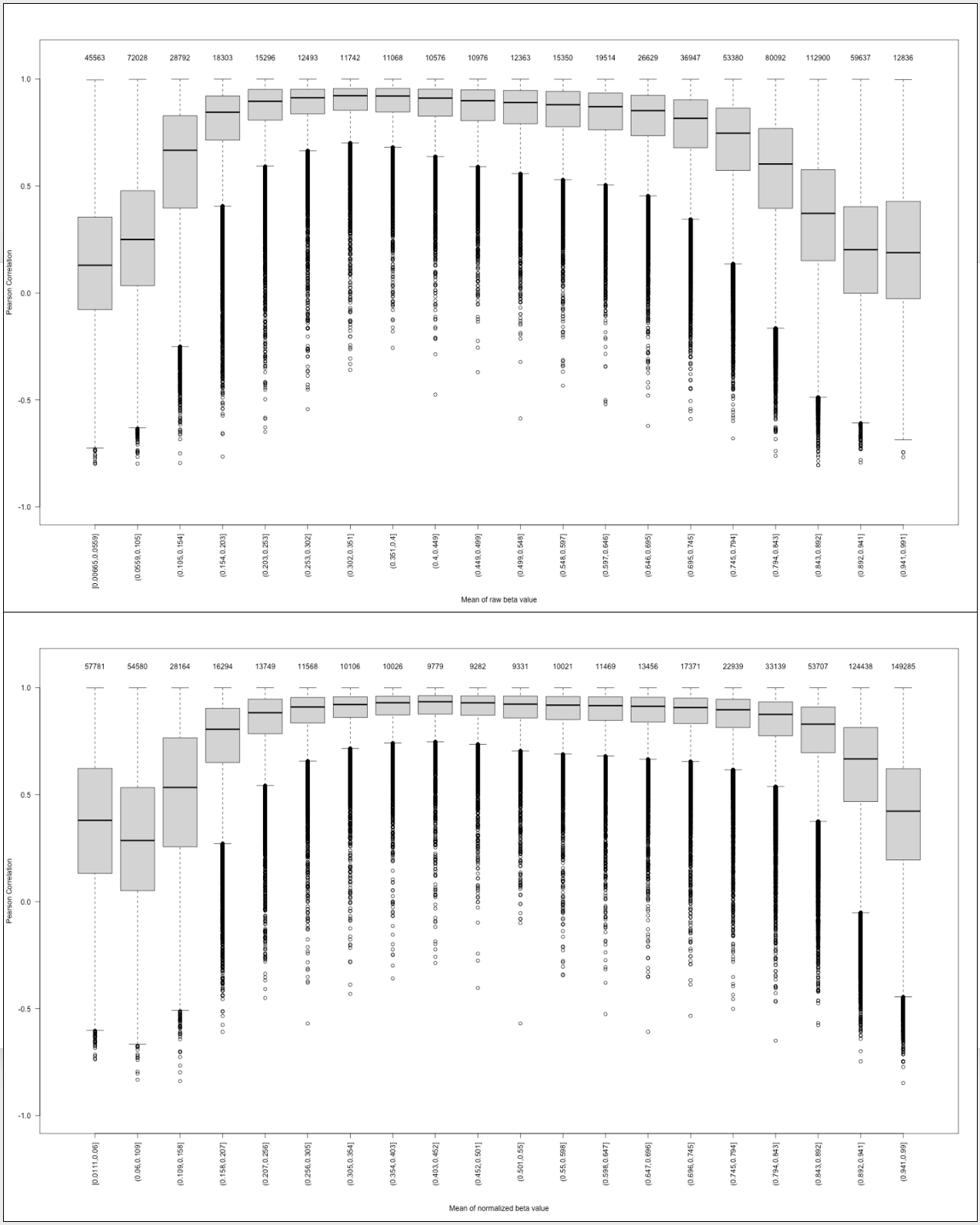
*

***Figure S9.*** *Equidistant plot of mean vs. Pearson’s correlation based on raw beta values (top) and SeSAMe 2 normalized beta values (bottom).* *Pearson’s correlation values are substantially lower when mean beta values are very low or very high (closer to 0 or 1) but improved following normalization with SeSAMe 2.*

*
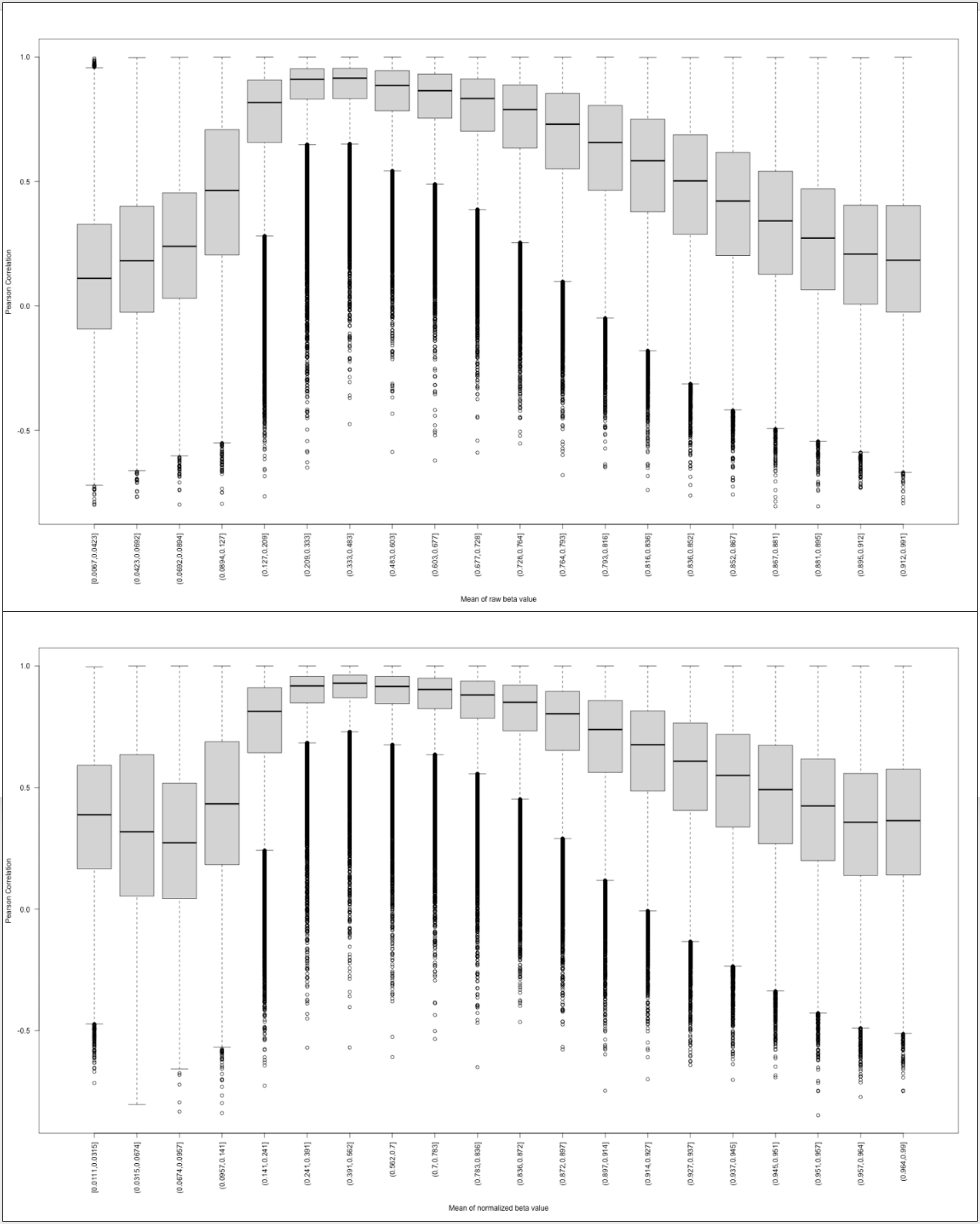
*

***Figure S10.*** *Percentiles plot of mean vs. Pearson’s correlation based on raw beta values (top) and SeSAMe 2 normalized beta values (bottom). Pearson’s correlation values are substantially lower when mean beta values are very low or very high (closer to 0 or 1) but improved following normalization with SeSAMe 2.*

*
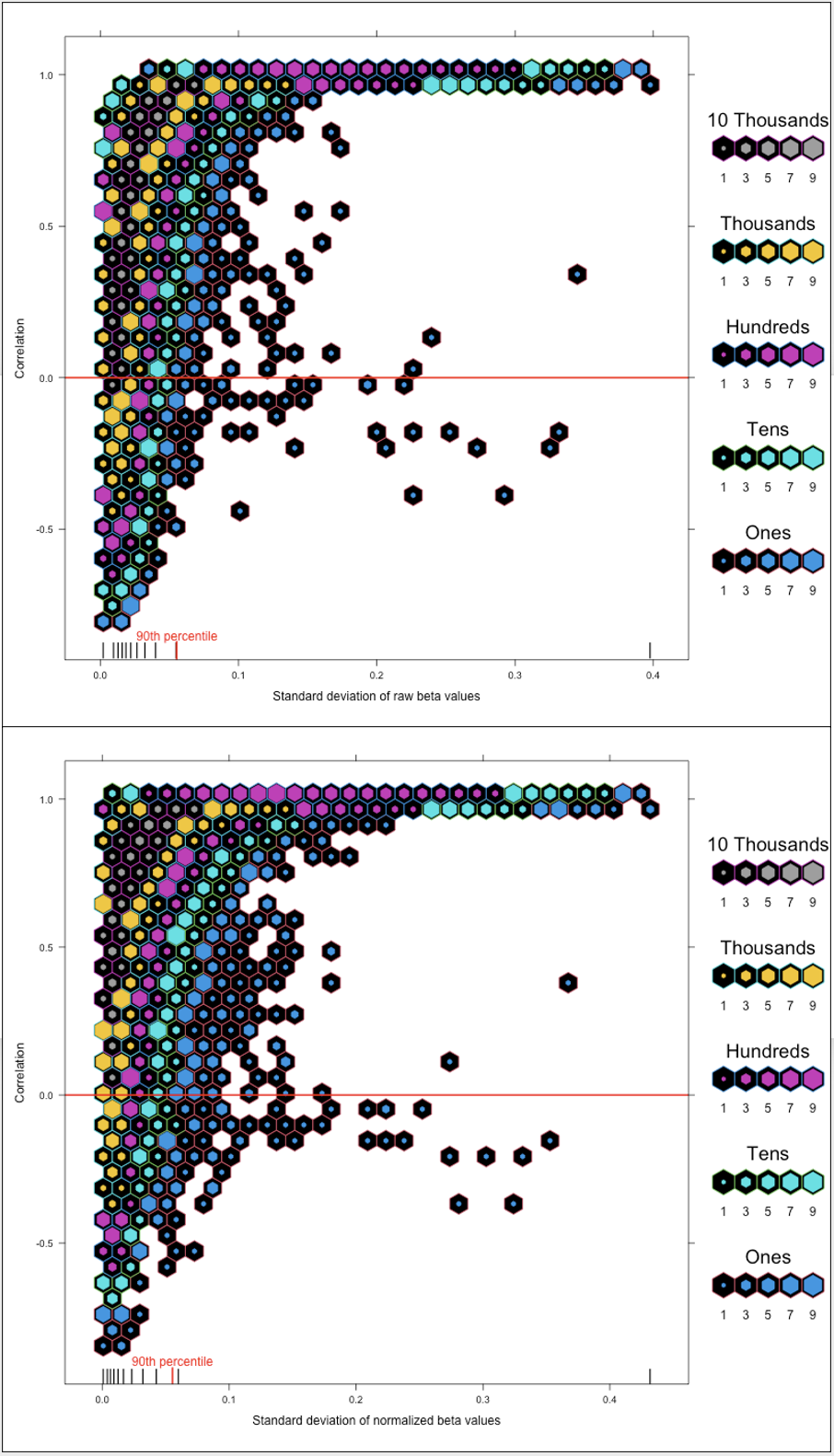
*

***Figure S11.*** *Hexabin scatter plot of SD vs. Pearson’s correlation based on raw beta values (top) and SeSAMe 2 normalized beta values (bottom). The majority of probes exhibit low SDs. Pearson’s correlation values are lower when the SDs of the beta estimates are low. Correlations improved following normalization with SeSAMe 2.*

*
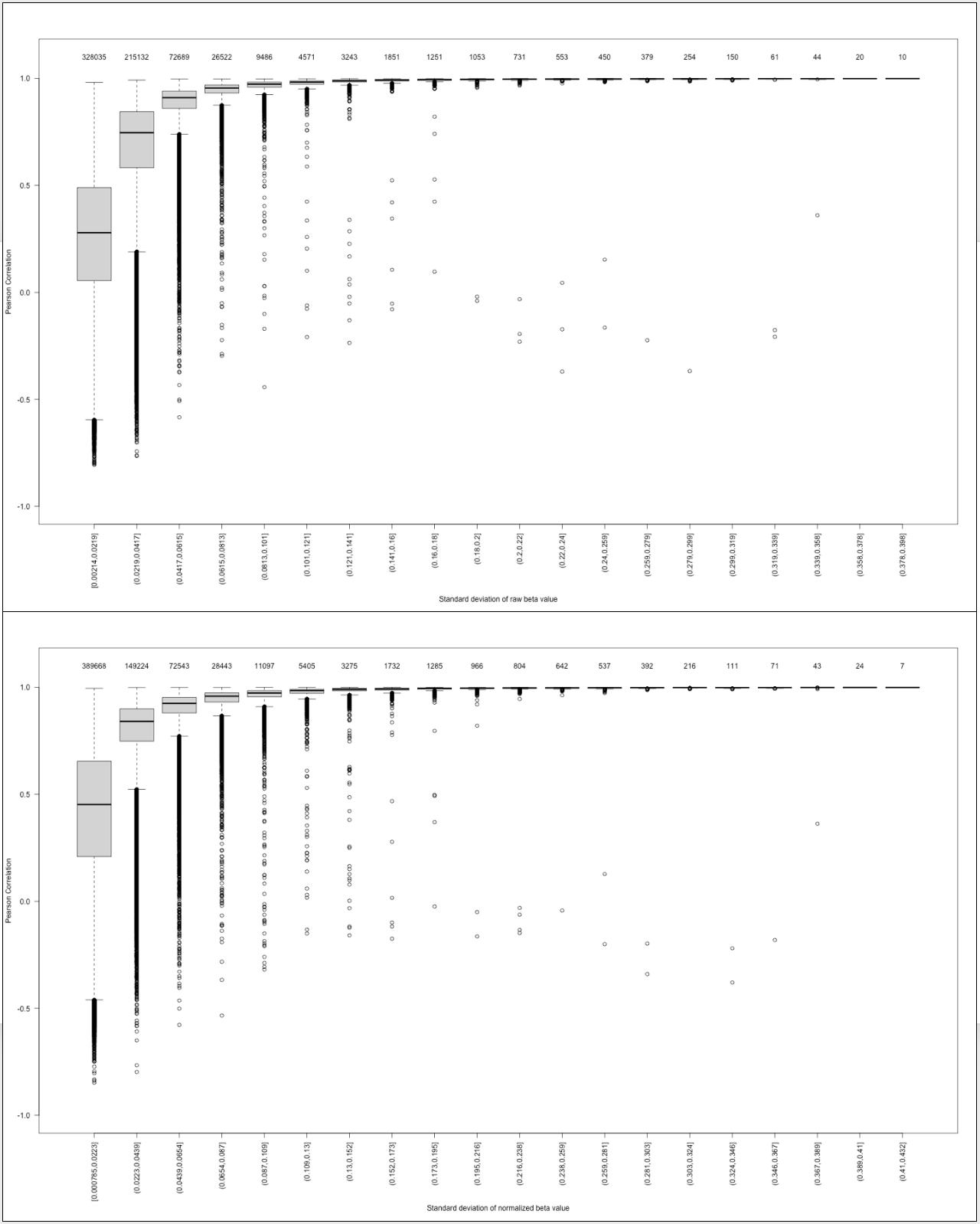
*

***Figure S12.*** *Equidistant plot of SD vs. Pearson’s correlation based on raw beta values (top) and SeSAMe 2 normalized beta values (bottom). The majority of probes exhibit low SDs. Pearson’s correlation values are lower when the SDs of the beta estimates are low.* *Correlations improved following normalization with SeSAMe 2.*

*
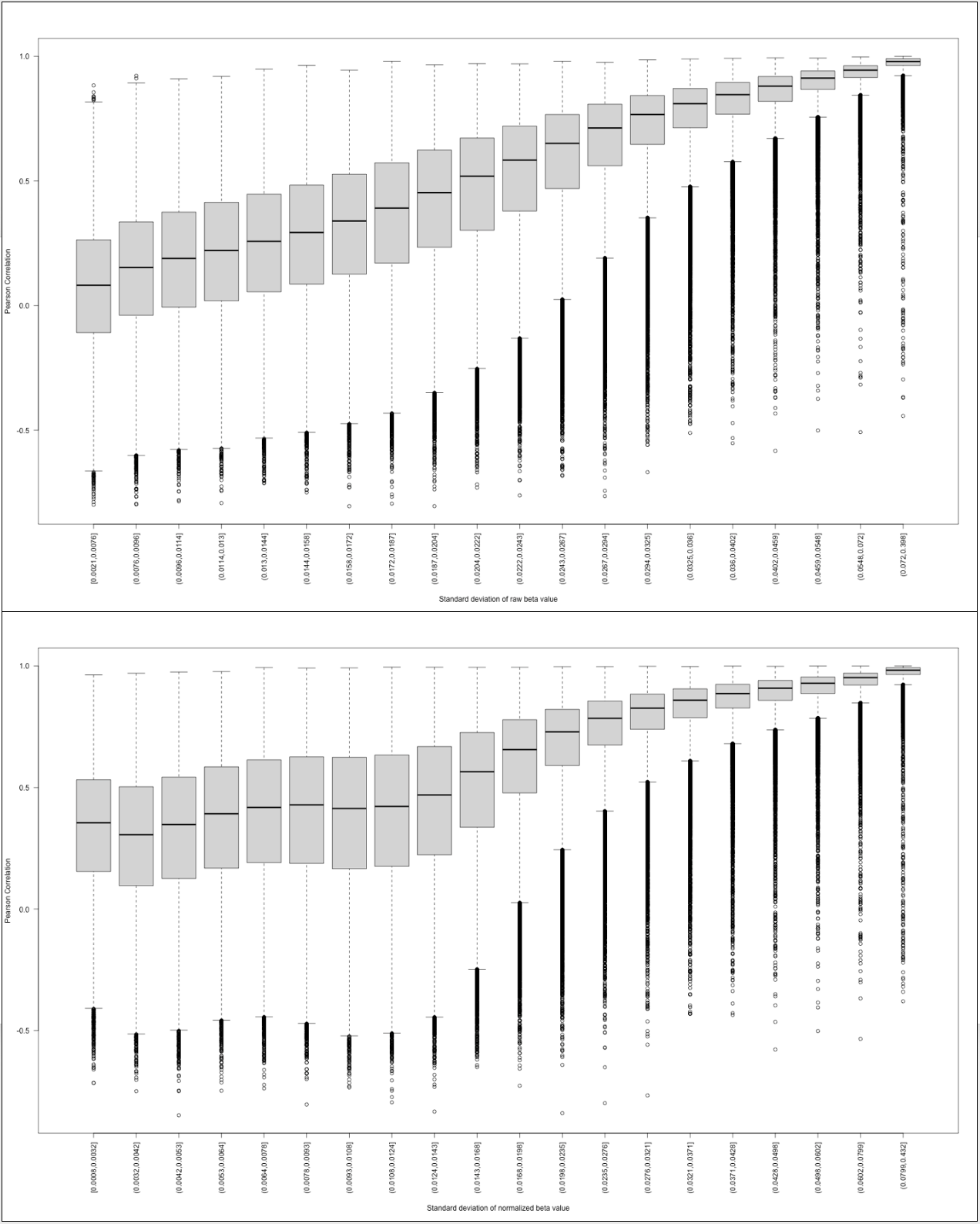
*

***Figure S13.*** *Percentiles plot of SD vs. Pearson’s correlation based on raw beta values (top) and SeSAMe normalized beta values (bottom). The majority of probes exhibit low SDs. Pearson’s correlation values are lower when the SDs of the beta estimates are low.* *Correlations improved following normalization with SeSAMe 2.*

*
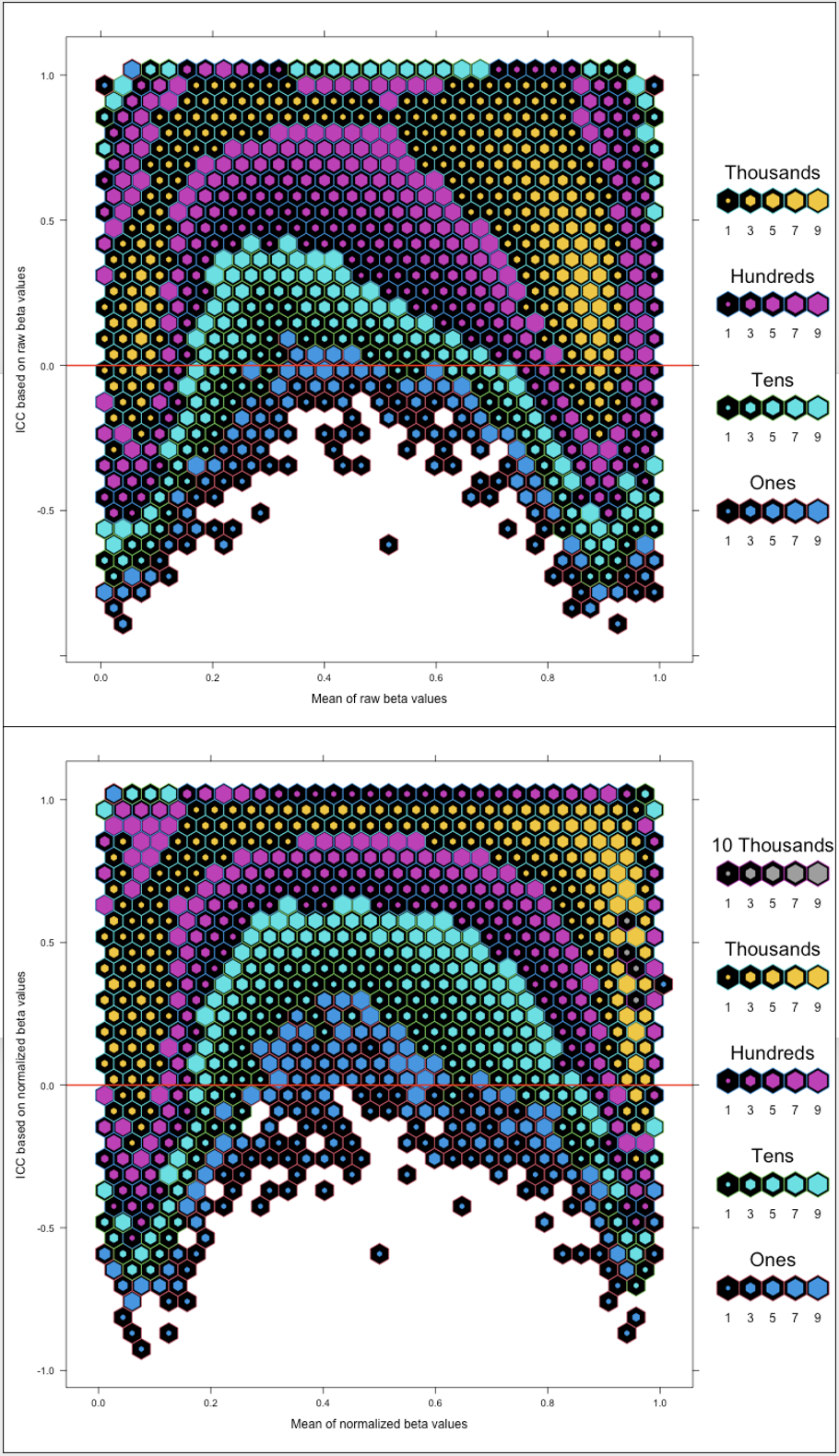
*

***Figure S14.*** *Hexabin scatter plot of mean vs. ICC based on raw beta values (top) and SeSAMe 2 normalized beta values (bottom). ICC values are substantially lower when mean beta values are low or high (close to 0 or 1). ICC values improved following normalization with SeSAMe 2.*

*
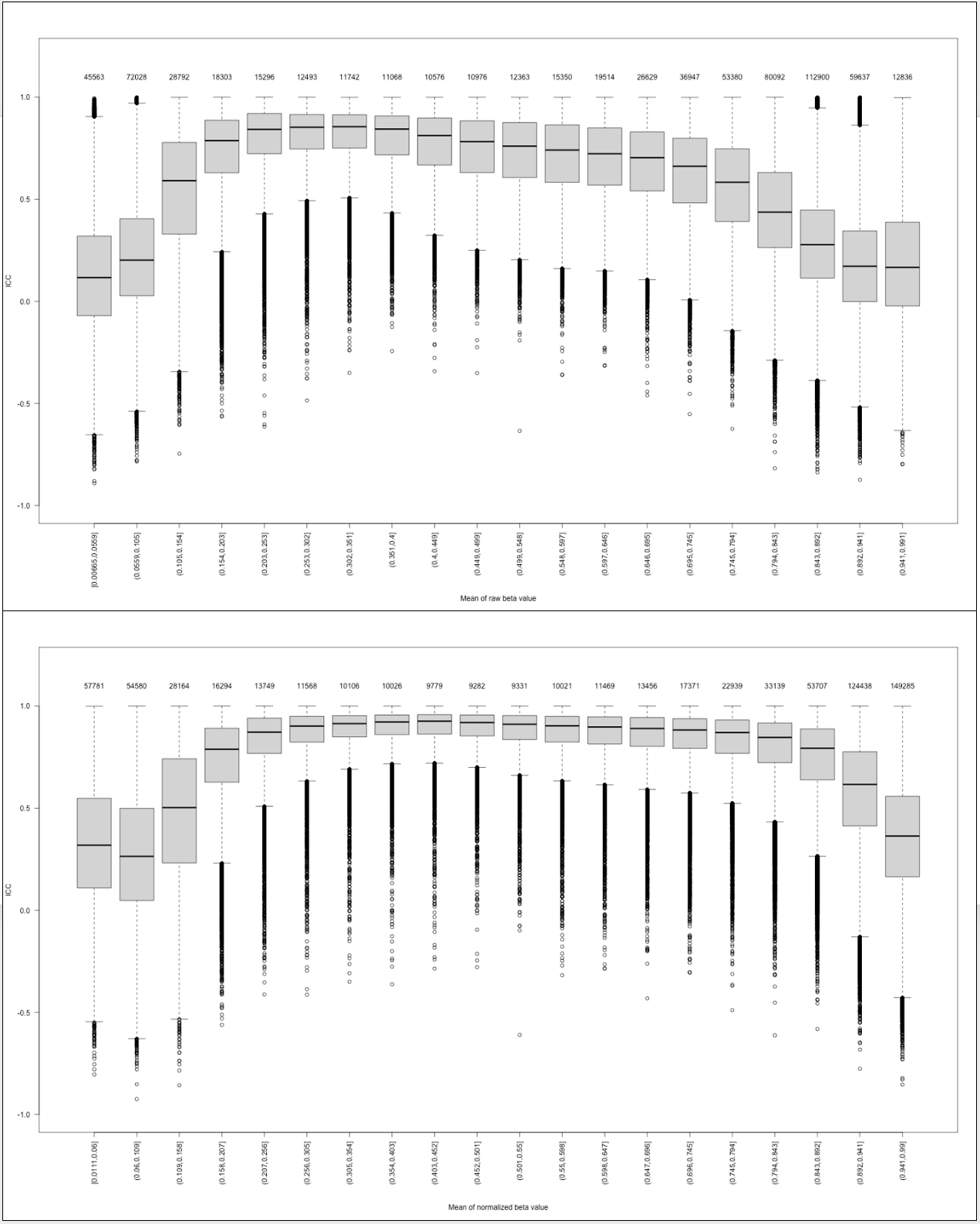
*

***Figure S15.*** *Equidistant plot of mean vs. ICC based on raw beta values (top) and SeSAMe 2 normalized beta values (bottom). ICC values are substantially lower when mean beta values are low or high (close to 0 or 1). ICC values improved following normalization with SeSAMe 2.*

*
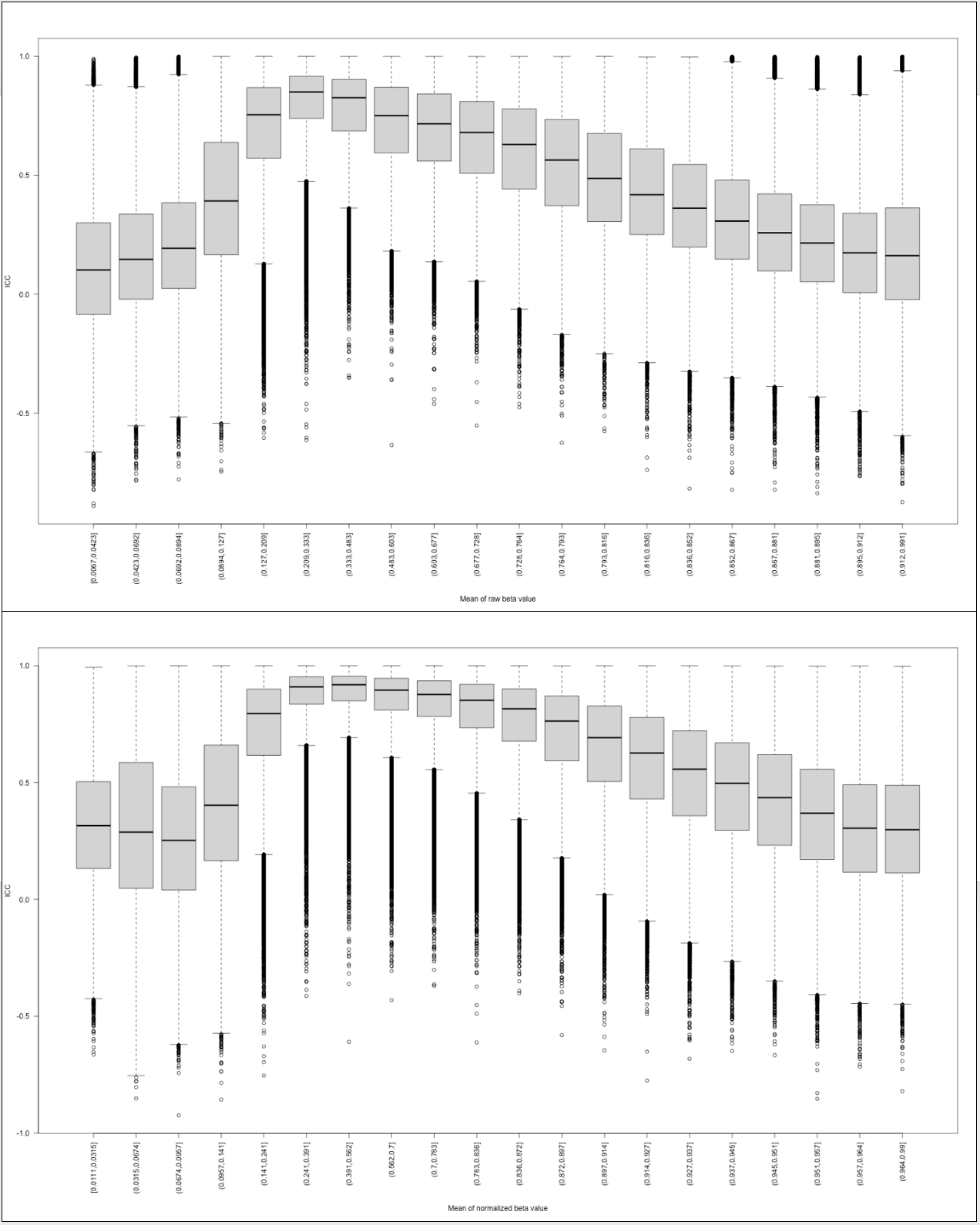
*

***Figure S16.*** *Percentiles plot of mean vs. ICC based on raw beta values (top) and SeSAMe 2 normalized beta values (bottom). ICC values are substantially lower when mean beta values are low or high (close to 0 or 1). ICC values improved following normalization with SeSAMe 2.*

*
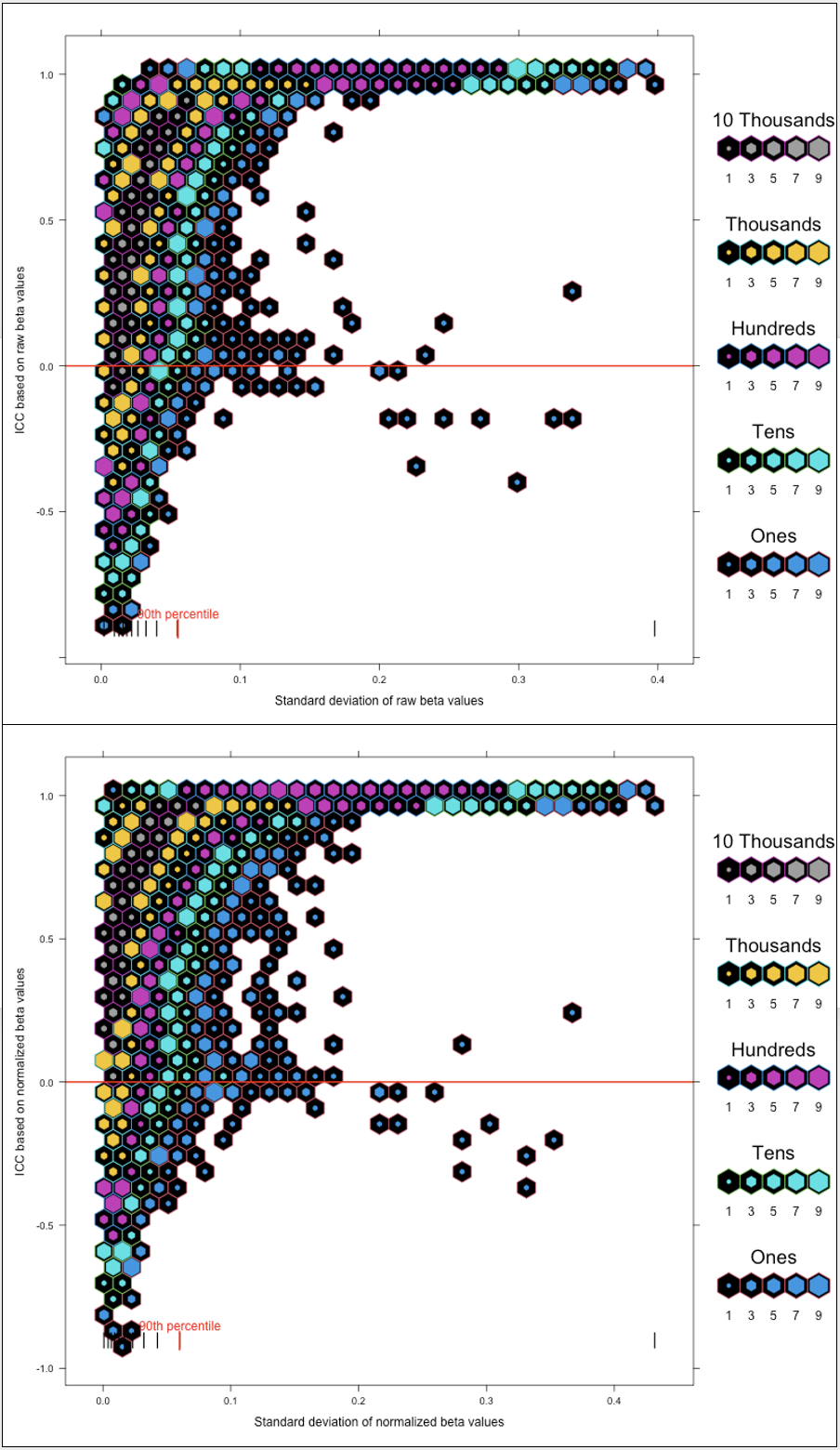
*

***Figure S17.*** *Hexabin scatter plot of SD vs. ICC based on raw beta values (top) and SeSAMe 2 normalized beta values (bottom). ICC values are lower when the SDs of the beta estimates are low. ICC values improved following normalization with SeSAMe 2.*

*
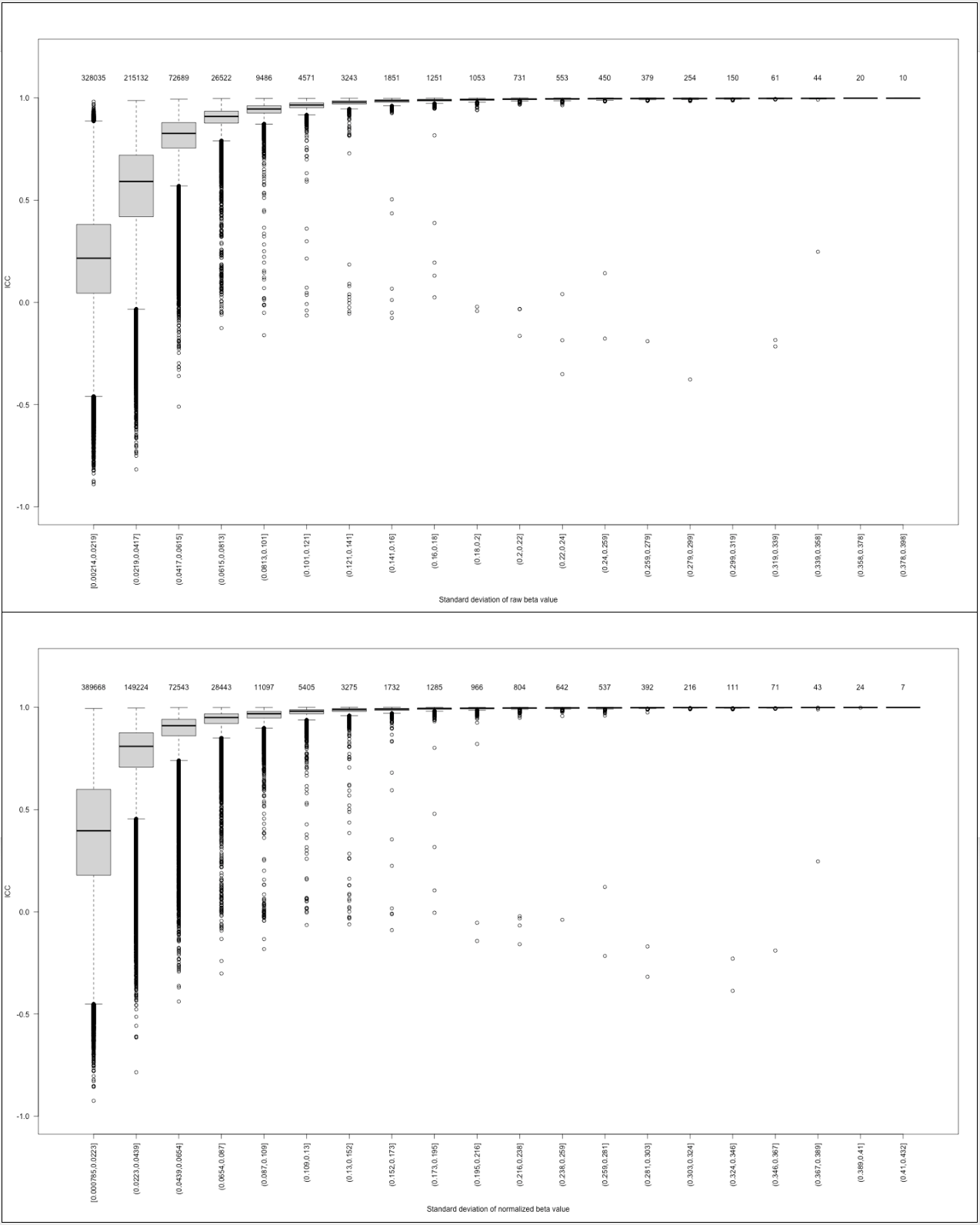
*

***Figure S18.*** *Equidistant plot of SD vs. ICC based on raw beta values (top) and SeSAMe 2 normalized beta values (bottom). ICC values are lower when the SDs of the beta estimates are low. ICC values improved following normalization with SeSAMe 2.*

*
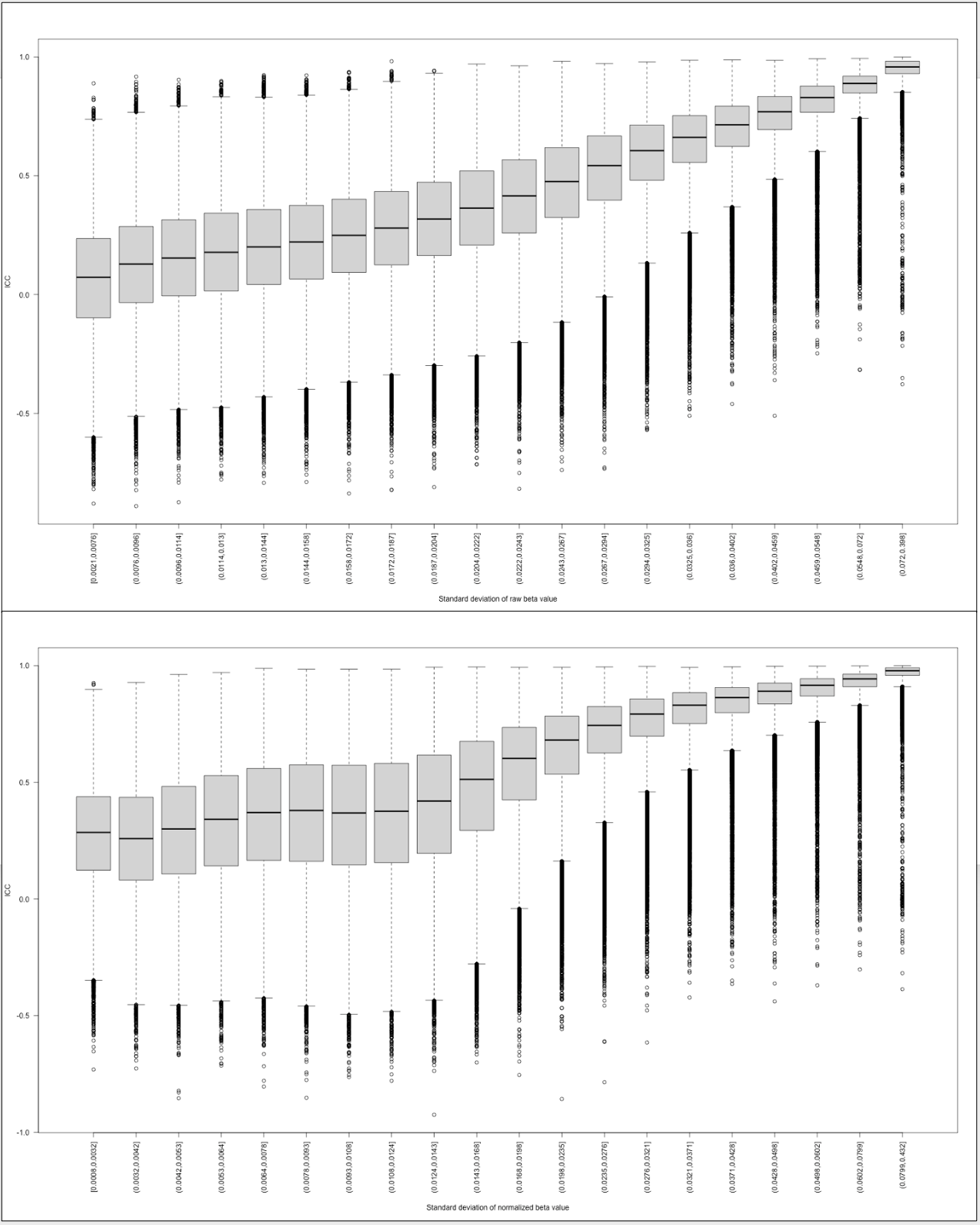
*

***Figure S19.*** *Percentiles plot of SD vs. ICC based on raw beta values (top) and SeSAMe 2 normalized beta values (bottom). ICC values are lower when the SDs of the beta estimates are low. ICC values improved following normalization with SeSAMe 2.*
